# Neighboring Alkali Cations as an Efficient Strategy for N_2_ Activation: A DFT Analysis

**DOI:** 10.3390/ijms27031311

**Published:** 2026-01-28

**Authors:** Jean C. Villa-Arpi, Romel Guañuna, Juan P. Saucedo-Vazquez, Thibault Terencio

**Affiliations:** 1 Departament de Ciència de Materials i Química Física Institut de Química Teòrica i Computacional, Universitat de Barcelona, c/Martí i Franquès 1-11, 08028 Barcelona, Spain; jvillaar32@alumnes.ub.edu; 2 CATS INVESTIGATION GROUP (CAtalysis Theory & Spectroscopy), Yachay Tech, Urcuqui 100119, Ecuador; romel.guanuna@yachaytech.edu.ec (R.G.); jsaucedo@yachaytech.edu.ec (J.P.S.-V.); 3 School of Chemistry and Engineering, Yachay Tech, Urcuqui 100119, Ecuador

**Keywords:** dinitrogen, activation, non-covalent, ions, DFT, polarization, alkali, alkaline-earth, magnesium, catalysis

## Abstract

Nitrogen gas is one of the most abundant resources on Earth, serving as a fundamental component in both biological and industrial processes. Nevertheless, this simple molecule can only be activated by a limited group of microorganisms in nature. Significant efforts have been devoted to replicating this biological activity using metalorganic approaches. However, it is becoming increasingly evident that non-covalent interactions, particularly ionic interactions, can further enhance catalytic reactions. In this work, the effect of alkali and alkaline-earth cations on dinitrogen activation was assessed using Density Functional Theory (DFT) at distances ranging from 2 to 10 Å. This analysis revealed three distinct activity regimes. In Case I, the polarization of the N_2_ molecule is the primary driving force; in Case II, the polarization effect is less pronounced; and in Case III, electrostatic interactions dominate, enhancing electron delocalization within the N_2_–M^n+^ system. Among the various cations, those belonging to group II-A are particularly noteworthy due to their high ionic potential and polarizing power, with Mg^2+^ standing out for its superior activity at an N_2_–Mg^2+^ distance of 2.7 Å. Consequently, these theoretical insights can serve as a guiding strategy for designing efficient N_2_-activating complexes that integrate covalent and non-covalent interactions synergistically.

## 1. Introduction

The nitrogen molecule (N_2_) is one of the most prevalent gases on Earth, accounting for approximately 78% of the atmosphere [1,2]. Its abundance provides compelling evidence that this molecule has played a pivotal role in numerous processes throughout the history of life on our planet. The most notable example is the nitrogen cycle, particularly nitrogen fixation [3,4,5]. These processes supply the fundamental building blocks for the synthesis of all biological structures, including the nitrogenous bases of DNA and the amino acids that form proteins [6,7]. Nitrogen gas is also a key precursor in the production of ammonia (NH_3_), the primary raw material used globally for fertilizers, whether through biological or artificial processes [6]. This resource is of growing interest for the emerging “nitrogen economy,” which focuses on energy production and hydrogen transport [8].

Although nitrogen appears to be an abundant resource, its direct utilization by biological organisms is not feasible due to its chemical inertness [9]. The low reactivity of the dinitrogen molecule arises from both thermodynamic and kinetic constraints. Thermodynamically, the molecule is hindered by its bond strength (945 kJ mol^−1^), negative electron affinity (−1.8 eV), and high ionization energy (15.0 eV). Additionally, N_2_ exhibits a substantial gap between the highest occupied molecular orbital (HOMO) and lowest unoccupied molecular orbital (LUMO) with a value of 22.9 eV, underscoring the molecule’s stability [10]. From a kinetic perspective, the poor electrophilicity and nucleophilicity of N_2_, combined with its lack of a permanent dipole, further limits its reactivity [11]. These properties all stem from the high symmetry of the molecule. Biologically, N_2_ can only be converted into useful nitrogen derivatives by specialized microorganisms, e.g., bacteria (Cyanobacteria, Azotobacter, and Rhizobium), fungi (Glomeromycota in Arbuscular mycorrhizal), and archaea (Methanococcus thermolithotrophicus), that possess a specific enzyme known as nitrogenase [12,13,14,15,16]. Consequently, nitrogen inputs into ecosystems are constrained by the distribution and abundance of these organisms [13,14,15]. To compensate for these limitations, substantial efforts have been devoted since the early 20th century for producing ammonia artificially. The Haber–Bosch (H–B) process remains the most widely employed method, synthesizing ammonia from nitrogen gas on potassium-promoted iron (or ruthenium) catalysts [17]. However, the H–B process is associated with high energy consumption and severe environmental impacts, motivating the search for alternative strategies.

One promising avenue involves designing catalysts inspired by biological systems, particularly biochemical components of nitrogenase [18]. This has led to the development of metalorganic complexes aimed at mimicking the enzyme’s ability to activate and reduce dinitrogen [19,20,21,22,23,24]. Despite significant progress, artificial nitrogen fixation still requires harsh conditions, and most metalorganic models have focused primarily on covalent interactions between N_2_ and transition-metal (TM) centers—a factor that limits catalytic efficiency.

Although TMs are essential for catalytic activity in enzymes, the effectiveness of biological machinery also relies heavily on second-sphere effects (i.e., non-covalent interactions) such as salt bridges, ligand positioning, and the presence of ions (e.g., cations and anions) [25]. The incorporation of cations into metalorganic complexes has attracted considerable attention in the last decade, especially in systems based on iron (Fe), molybdenum (Mo), uranium (U), and related metals [26,27,28,29,30,31,32,33]. These complexes are designed to reproduce the behavior of nitrogenase active sites, although comparing their effectiveness remains challenging since most represent experimental attempts to mimic biological structures. Examples include β-diketiminate iron complexes [26,27,28]; tris(phosphino)silane, borane, and alkyl ligand iron complexes [27]; (n-crown-P_3_-R)MoCl_3_ complexes [29]; pyrrolyl–pyridine iron complexes [31]; and siloxide uranium complexes [32,33], many of which feature side-on coordination of N_2_. Such studies suggest that catalysts become more efficient when assisted by cations, although the precise role of these species remains uncertain. Numerous transition metal–N_2_ complexes have been reported, featuring diverse metals, ligands, and bonding modes. In most cases, when alkali cations are present, they either engage in end-on interactions with the N_2_ σ-orbital—enhancing N_2_ polarization [34]—or in side-on interactions with the N_2_ π-orbitals [27].

In recent years, cations have gained new significance in catalysis through insights provided by the field of superatoms [35,36,37]. Such structures mimic the properties of isolated atomic or ionic species, providing a platform to explore cationic behavior without the practical limitations of working with free cations (IA and IIA elements) [38]. In particular, alkalis and superalkalis—characterized by their low ionization energies (IEs)—can reduce highly stable molecules such as CO_2_ and N_2_. These systems have highlighted the potential of cations in substrate activation and reduction, though further analysis is required to fully elucidate their role in N_2_ activation within metalorganic structures.

In this work, we present a theoretical study into how alkali and alkaline-earth metal cations (AMs) influence the activation of N_2_ through side-on interactions [38]. This binding mode has been recognized experimentally as one of the most promising configurations for weakening the N–N bond, particularly when two late transition metal centers cooperate with a third appended Lewis acid metal site [27,39]. By analyzing the variation in the dinitrogen–cation distance (N_2_–M^n+^), we aim to clarify the behavior of these ionic species and provide a theoretical foundation for the design of more efficient and environmentally sustainable catalysts for N_2_ reduction.

## 2. Results

The most critical parameters for activating the dinitrogen molecule through a catalyst are as follows: (i) the decrease in the N–N bond stretching frequency (ν_N–N_, 2359 cm^−1^ for free N_2_), (ii) the weakening of the N–N bond strength, and (iii) the elongation of the N–N bond (1.098 Å for atmospheric N_2_). These factors collectively reflect a higher degree of N_2_ reduction, driven by increased electron density in the N_2_ π* molecular orbitals.

### 2.1. Direct Influence of Cations on the Vibrational Frequencies and Bond Length of N_2_

**Figure 1:**
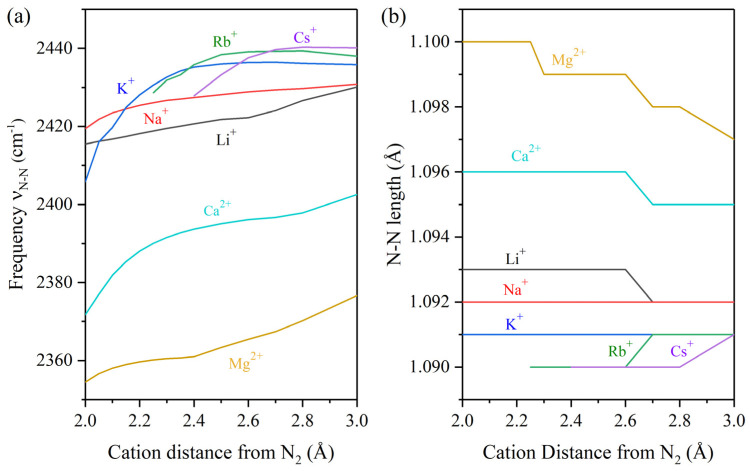
Analysis of (**a**) the vibrational frequency and (**b**) bond length at distinct M^n+^–N_2_ distances where M: Li, Na, K, Rb, Cs, Mg, Ca and n: 1 or 2 depending on the cation type.

As cations approach the N_2_ molecule (Figure 1), they exert a pronounced influence on the ν_N–N_, which has a reference theoretical value of 2445.93 cm^−1^. This observation provides clear evidence of a chemical interaction between the ions and the dinitrogen molecule. Simultaneously, the N–N bond length undergoes slight changes for all cations, although the magnitude of this variation remains relatively minor. This suggests that cationic species act primarily as auxiliary components of the main TM catalyst.

For divalent cations (e.g., Mg^2+^ and Ca^2+^), the N_2_–M^n+^ distance is directly proportional to the vibrational frequency and inversely proportional to the N–N bond length. In contrast, monovalent cations (Na^+^, Li^+^, K^+^, Rb^+^, Cs^+^) display weaker effects compared to alkaline-earth cations. Among them, Na^+^ and Li^+^ show the most pronounced activity, producing greater variations in the discussed parameters compared to K^+^, Rb^+^, or Cs^+^. These results are consistent with the expectation that stronger effects arise as the cation approaches more closely to the dinitrogen molecule. Nevertheless, it is essential to consider additional descriptors to fully elucidate the mechanisms and activities associated with each cationic species.

### 2.2. Analysis of the Mayer Bond Order (MBO) in the Dinitrogen Molecule

**Figure 2:**
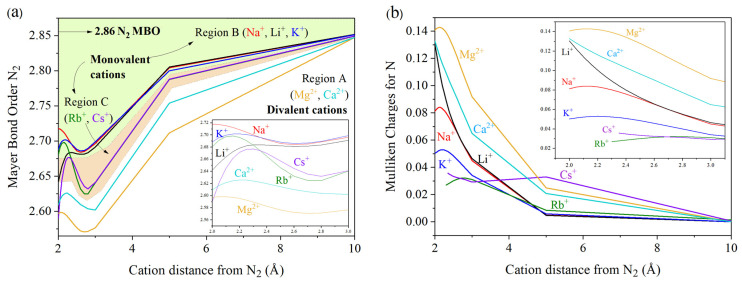
(**a**) MBO (N–N) variation for N_2_–M^n+^ systems from 2 to 10 Å, and (**b**) Mulliken Charges (MC) analysis for N at N_2_–M^n+^ distances from 2 to 10 Å where M: Li, Na, K, Rb, Cs, Mg, Ca and n: 1 or 2 depending of the cation type.

A baseline MBO (N–N) value of 2.86 was considered for the isolated N_2_ molecule. Different degrees of variation were obtained depending on the cationic species employed (Figure 2a). At large N_2_–M^n+^ distances, no meaningful changes could be identified. For all systems, however, a notable minimum is observed, where the lowest MBO (N–N) value is reached for each configuration. This minimum indicates a significant weakening of the N_2_ triple bond. Importantly, the effective N_2_–M^n+^ distance at which this weakening occurs appears to be independent of the specific cationic system. MBO values corresponding to N_2_–M^n+^ distances below 2 Å were excluded, as they break with the principles of van der Waals radii and the repulsion between atomic species.

A clear distinction emerges between monovalent and divalent cations. The latter induce a larger decrease in MBO relative to the baseline. For instance, in the cases of Mg^2+^ and Ca^2+^, effective N_2_–M^n+^ distances of 2.7 and 3.0 Å yield MBO (N–N) values of 2.5707 and 2.602, respectively (Figure 2a, region A). In contrast, the behavior of monovalent cations can be divided into two groups, designated as region B (Li^+^, Na^+^, K^+^) and region C (Rb^+^, Cs^+^). Region C shows a stronger MBO decrease (2.6247 and 2.6218, respectively) at effective N_2_–M^n+^ distances of 2.8 Å, whereas region B exhibits a more moderate reduction (2.6833, 2.6839, and 2.6859) at distances of 2.4, 2.6, and 2.6 Å, respectively. On average, divalent cations induce a 9.67% reduction in MBO (N–N), compared to 6.86% and 8.26% reductions for monovalent cations in regions B and C, respectively.

### 2.3. Analysis of Mulliken Charges Transferred to N_2_

Because cations are ionic species with a net charge (n^+^), their influence on the N_2_ electron density can also be examined through the Mulliken charges (MCs) of each chemical element. Since ionic interactions involve electron gain or loss [40], such effects must be reflected in the total MC of each element in the system. Conversely, if no change in the MC is observed between an ionic species and N_2_, this suggests a covalent interaction in which electrons are shared rather than transferred. Figure 2b illustrates the inverse proportionality between MC(N_2_) and N_2_–M^n+^ distances, indicating stronger electron transfer (i.e., ionic behavior) at shorter N_2_–M^n+^ separations. One factor contributing to the decrease in MBO (N–N) shown in Figure 2b is the ability of cationic species to promote electron transfer to the nitrogen atoms. As a result, a portion of the electron density originally associated with the N_2_ triple bond (i.e., covalent interaction) is transferred to an ionic interaction with the cation, thereby weakening the N–N bond order.

From the N_2_–M^n+^ MC plot, divalent cations are seen to induce greater electron transfer. Within this group, Mg^2+^ is particularly notable. In general, the N_2_ triple bond weakens as electron transfer from the cation to the nitrogen atoms increases. However, to fully disentangle the contributions of MBO and MC, additional concepts must be introduced.

The ionic potential (*μ*) is defined as the ability of ions or charged atoms to attract (or repel) electron clouds [41]. A related property is the polarizing power (*ϕ*), which describes a cation’s capacity to distort the electron density of an anion or another molecule [41]. Complementary to these is polarizability (*α*), which measures the extent to which a chemical entity can be deformed by an electric field [41,42]. As shown in Table 1, *α* decreases when *μ* or *ϕ* increase, and *α* is directly proportional to the ionic radius of the cation.

**Figure A1:**
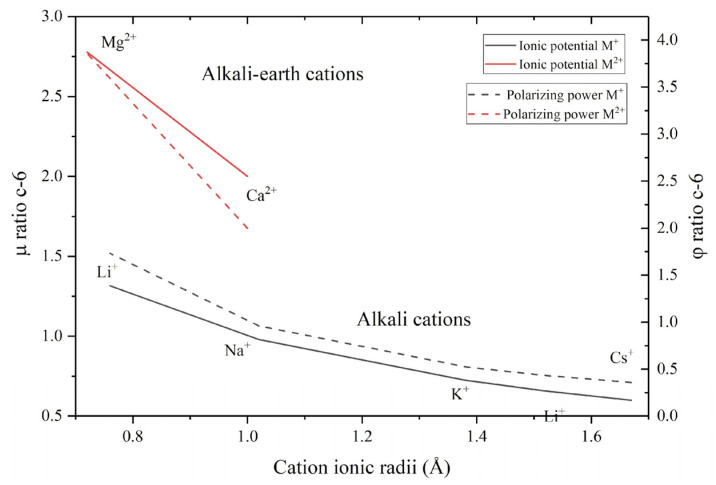
Variation in the ionic potential (μ) and polarizing power (ϕ) of alkali and alkaline-earth cations.

Higher MC(N_2_) values are obtained at shorter N_2_–M^n+^ distances. At these separations, Mg^2+^ and Ca^2+^ stand out from the other species. Among the monovalent cations, Li^+^ and Na^+^ display higher MC values than the larger alkali cations (K^+^, Rb^+^, Cs^+^). This behavior can be attributed to ionic potential, which is higher for highly charged cations with small ionic radii, such as the early-group alkaline cations (Table 1 and Figure A1).

### 2.4. Raman Spectroscopy as an Indicator of Dinitrogen Polarization

**Figure 3:**
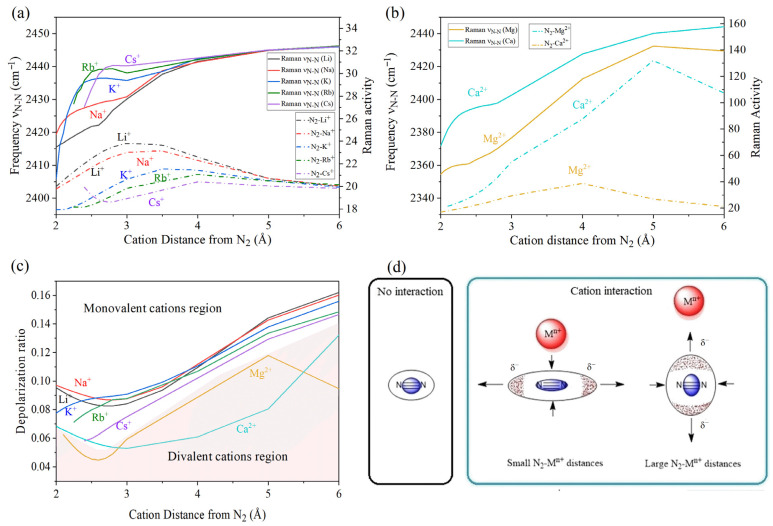
Raman vibrational frequency and Raman activity calculations for M^n+^–N_2_ systems at distances from 2 to 6 Å: (**a**) monovalent cations; (**b**) divalent cations. (**c**) Results of depolarization ratio obtained from calculations of M^n+^–N_2_ systems at distances from 2 to 6 Å. (**d**) Cation interactions at small and large distances with dinitrogen.

Additional insights can be obtained from Raman spectroscopy simulations. The Raman ν_N–N_ frequencies typically follow a trend similar to that observed for IR vibrational frequencies. Divalent cations induce more pronounced changes in vibrational frequencies compared to monovalent cations (Figure 3a,b). However, all cations exert a weakening effect on the N≡N triple bond (2445.93 cm^−1^, theoretical reference), as reflected by this common experimental descriptor. In principle, all cations are expected to decrease the ν_N–N_ value in a relatively uniform manner. Indeed, cations with high ionic potential and electronegativity (e.g., Mg^2+^, Ca^2+^, Li^+^, Na^+^) show comparable trends. In contrast, larger cations display an alternating pattern, with stronger frequency reductions observed at shorter N_2_–M^n+^ distances. Nevertheless, these latter cases are less reliable, since they imply that their massive cationic species are near the electron density of N_2_, leading to the repulsion of the chemical species in experimental conditions—a situation that is experimentally unstable.

Computational modeling also provides additional parameters such as the Raman activity (RA) and the depolarization ratio (τ). The former, expressed in units of Å^4^/amu, reflects the characteristic Raman signal intensity. Specifically, Raman intensity depends on polarizability and molecular symmetry, thereby providing evidence for bonding covalency and structural features. The depolarization ratio, on the other hand, is defined by the following equation:

(1) $$\tau =\frac{I_{⊥}}{I_{∥}}=\frac{I_{depolarized}}{I_{polarized}}$$

where the symbols $I_{⊥}$ and $I_{∥}$ represent the perpendicular and parallel components of Raman scattered light, respectively. This parameter allows for the distinction of two regions: if the value of *τ* is less than 0.75, the molecule is considered polarized, whereas values above this threshold correspond to depolarized bands. In general, all cations induce larger changes in RA (Figure 3a,b) compared to the isolated N_2_ molecule (19.559), which again highlights the polarizing power of these species. For the case of *τ* with a base value of 0.1743, variations are evident across the entire range of distances considered, further confirming the strong polarizing effect of the cations. Moreover, an inverse proportionality between RA (Figure 3a,b) and *τ* is observed (Figure 3c), consistent with the definition of *τ* (lower polarization ratios correspond to higher overall system polarization).

For N_2_–M^+^ interactions, the maximum RA occurs in the 3–4 Å range, shifting toward larger distances as the cation size increases. A similar trend is observed for N_2_–M^2+^ interactions, although the peak appears in the 4–5 Å range. For a chemical species to exhibit RA, it must undergo a change in polarizability, which can be defined as the relative tendency of an electron cloud to be distorted from its equilibrium distribution.

### 2.5. Comparison of Methods

When the present results for the N_2_–M^n+^ interactions (M = Mg, Li, and Cs) are compared with other computational methods such as CAM-B3LYP, M06-2X, and CCSD(T), a slight decrease in the N–N bond order is observed as the cation approaches (i.e., at shorter N_2_–M^n+^ distances). This reduction indicates a weakening of the N–N bond, primarily due to charge polarization or back-donation effects induced by the cationic electric field.

**Figure 4:**
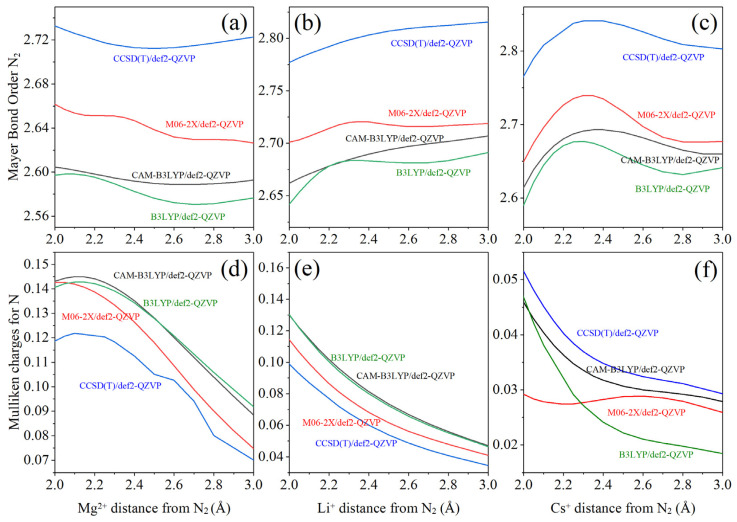
Validation of the results with other methodologies for Ca^2+^–N_2_, Li^+^–N_2_ and Cs^+^–N_2_ systems taking as reference the (**a**–**c**) MBO (N–N) variation for M^n+^–N_2_ systems from 2 to 3 Å; and (**d**–**f**) Mulliken Charges (MC) analysis for N at M^n+^–N_2_ distances from 2 to 3 Å where M: Mg, Li, Cs and n: 1 or 2 depending of the cation type.

The CCSD(T) method (Figure 4) predicts a modest weakening of the N≡N triple bond, while still preserving general trends similar to those obtained with B3LYP. For the N_2_–Mg^2+^ interaction (Figure 4a), the bonding is mainly electrostatic (ionic polarization) with minimal orbital overlap, given the bare variation in the MBO. Functionals containing a higher fraction of exact exchange, such as M06-2X, more closely resemble the behavior predicted by CCSD(T). However, the CCSD(T) trend also reveals a slightly greater MBO reduction near the minimum of the potential curve, consistent with the qualitative behavior observed using the B3LYP functional.

In the case of the N_2_–Li^+^ interaction (Figure 4b), the MBO decreases moderately as Li^+^ approaches the dinitrogen molecule. At longer distances, B3LYP tends to overestimate the polarization-induced weakening, reflecting its tendency to over-delocalize the electronic density.

For the N_2_–Cs^+^ system (Figure 4c), all methods reproduce the general trend predicted by CCSD(T), indicating that Cs^+^ exhibits its most pronounced influence on N_2_ within the range of 2.8–3.0 Å. At this distance, B3LYP accurately reproduces the subtle interaction between Cs^+^ and N_2_.

Regarding the Mulliken charges (Figure 4d–f), B3LYP closely follows the behavior predicted by CCSD(T) and other functionals, confirming the polarization of the dinitrogen molecule and supporting the interpretation of a partial N_2_ activation upon interaction with the cations. Besides the limitations of B3LYP functional, its results are meaningful and can be used to explore cationic behaviors.

### 2.6. Other Descriptors of the Interaction

#### 2.6.1. Interaction Energies

The enthalpy of the interaction of N_2_ with the cation and the respective N_2_ bond dissociation energy were calculated (Table 2). All values were obtained from the optimized geometries of the complexes (2–3 Å for respective cations), ensuring that the computed energetic descriptors correspond to the most stable configurations at the chosen level of theory.

The interaction energies vary between 11.80 for Cs^+^ and −118.99 kJ/mol for Mg^2+^, following the strength of the polarizing power. The presence of the cation induces a decrease in N_2_ bond dissociation energy from 945.35 kJ/mol for free N_2_ to a minimum of 826.36 kJ/mol for Mg^2+^, showing a partial activation correlating with the charge concentration of the cation. The primal effect on the bond is given by the change in electrostatic interactions.

Thermodynamically, the data reveals a contrast between Enthalpy (ΔH) behavior. The ΔH becomes increasing with more polarizing cations, showing the side on coordination is energetically favorable. Also, it facilitates electron density distribution toward the metal center, promoting enthalpic activation.

#### 2.6.2. Comparison of Population Analysis

Positive values indicate that nitrogen acts as a partial charge acceptor, with the magnitude of this charge serving as an indicator of the efficiency of electron transfer from the metal. The atomic charges of the metals in the N_2_–M^n+^ reflect the loss of the charge localization on the cation (Table 3). Among the alkali metals, a progressive reduction in the atomic charges of nitrogen is observed as one descends the group (Li → Cs), with values ranging from 0.057 (Li, Mulliken) to 0.017 (Cs, Mulliken). This trend, consistent across Mulliken and Löwdin, directly correlates with the increase in ionic radius and the decrease in electronegativity down the group. In the Hirschfeld method appears a discrepancy where K and Rb describe higher charges on N. All charge schemes indicate that alkaline-earth metals induce a higher charge on N. Mulliken and Hirshfeld display close values whereas Lowdin finds a higher charge on Ca. All charge schemes describe the same general trend but slight differences appear depending on how is defined the partition of the electronic cloud, suggesting that there are differences in the repartition of the electrons more subtle than just the value of the charges.

#### 2.6.3. Different Bond Order Schemes

For all the cations studied, the values of bond order (Table 4) remain inferior to the free nitrogen molecule and high across the four theoretical methods employed (minimum value: 2.599 for Mg, Mayer) reflecting that these cannot be the main catalyst but can only help other more efficient catalysts, such as transition metals.

The heavier metals in the group I (K, Rb, Cs) tend to show slightly higher bond orders in the Löwdin/Wiberg, Laplacian and Mayer methods, whereas Fuzzy analyses suggest that all cations have a similar effect. This trend suggests that the polarizing power of the ion is the main cause except in the Fuzzy case which captures a different phenomenon. The lowest bond orders are found for group II cations, and specifically for Mg^2+^ (2.599, Mayer) also confirming that the electrostatic charge is particularly important as a main component of the interaction between cation and N_2_ molecule. The different behavior of Fuzzy atoms theory is very interesting as it is a theory which uses a different partition of the atoms, suggesting that more traditional bond order definitions are unable to capture the whole physical description of the interaction.

#### 2.6.4. The Quantum Theory of Atoms in Molecules (QTAIM) Analysis

**Figure 5:**
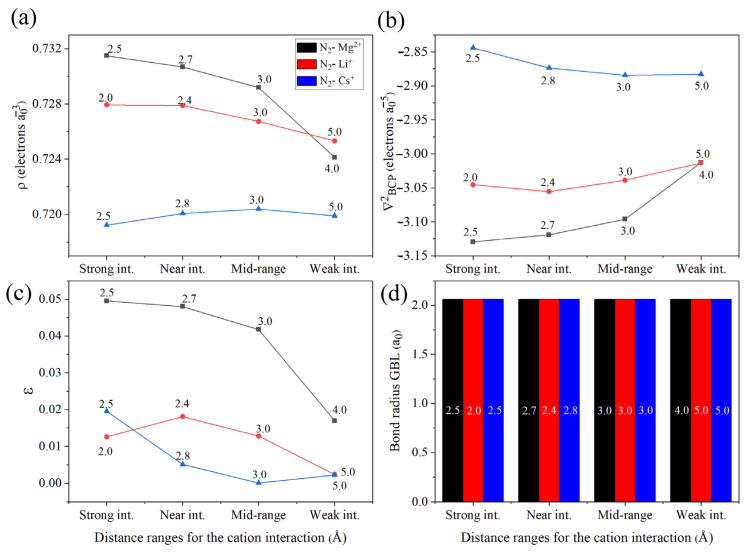
QTAIM properties analysis for the (3, −1) BCP N2 N1 point. (**a**) Variation in the electron density, (**b**) variation in the Laplacian, (**c**) variation in the ellipticity, and (**d**) variation in the bond radius for N_2_–M^n+^ systems at the range of 2–3 Å where M: Mg, Li, Cs and n: 1 or 2 depending of the cation type.

For a deeper understanding of the interaction nature, the wavefunctions of the N_2_–M^n+^ systems (M = Mg, Li, and Cs) within the 2.0–3.0 Å range were analyzed using the QTAIM framework. The graphical representations as well as miscellaneous properties of these models are provided in Tables S1–S6 and Figure S1. The key feature of interest is the bond critical point (BCP) located between the two nitrogen atoms, defined as the (3, −1) BCP N_2_–N_1_ point (Figure S1). Several topological properties were extracted from this point, including the electron density (*ρ*), the Laplacian of the electron density ($\nabla_{BCP}^{2}$), the ellipticity (*ε*), and the bond radius (Figure 5).

In Figure 5a, both Mg^2+^ and Li^+^ exhibit similar behavior, where *ρ* decreases as the cation–N_2_ distance increases, indicating a weakening of the N≡N bond with reduced electrostatic influence. In contrast, for Cs^+^, *ρ* decreases as the cation approaches, suggesting a different polarization pattern dominated by diffuse charge distribution. Regarding $\nabla_{BCP}^{2}$ (Figure 5b), the Mg^2+^ and Li^+^ systems show increasingly negative values as the cation approaches the N_2_ molecule, consistent with enhanced electron density concentration in the bonding region.

For the ellipticity (Figure 5c), the values slightly increase as the cations approach the N_2_ molecule. Although the absolute values remain small, this trend reflects a minor distortion of the π-electron cloud, confirming that the interaction is primarily electrostatic in nature, with subtle variations in magnitude depending on the cation. Since ε increases as the cations approach N_2_ in the cases of Mg^2+^ and Cs^+^, which is also signal of the π nature of the bond, the most favorable coordination distances for these cations lie near the previously proposed values of 2.7 and 2.8 Å (see Section 2.2). In contrast, Li^+^ can effectively interact with N_2_ even below 2.0 Å, owing to its small ionic radius, which allows for a stronger local field and thus a higher capacity to induce polarization and partial activation of N_2_.

Finally, Figure 5d shows the variation in the bond radius for this family of systems. The bond radius remains nearly constant across all cases, indicating that the primary role of the cations is to perturb the electron density distribution rather than to induce significant structural distortions in the dinitrogen molecule.

**Figure 6:**
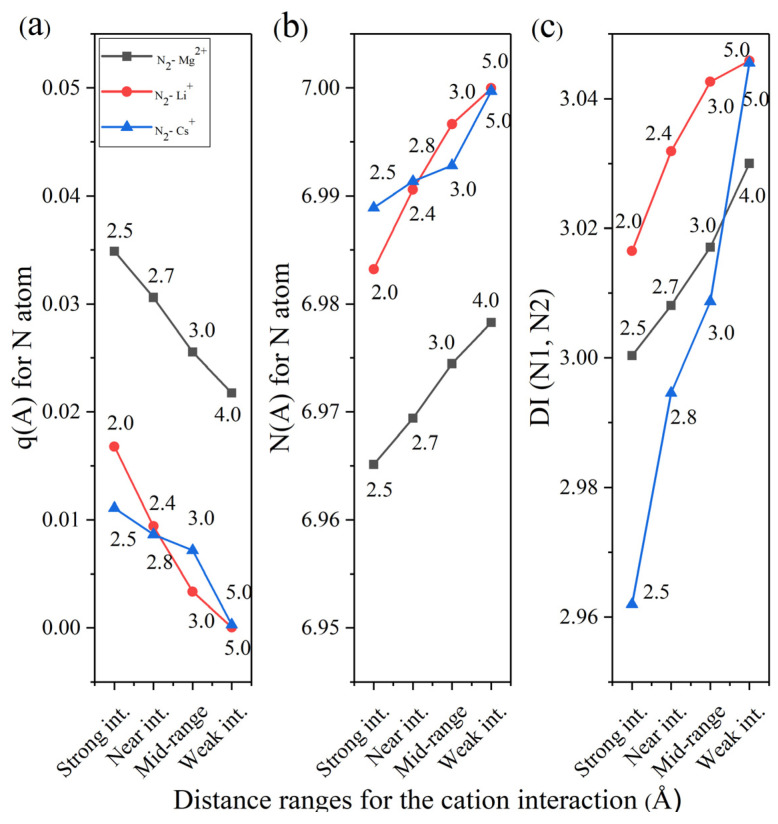
QTAIM-derived properties from the integration of the N atomic basin. (**a**) Atomic charges for the N atom, (**b**) number of electrons for the N atom, and (**c**) delocalization index (DI) between N nuclei for N_2_–M^n+^ systems at the range of 2–3 Å where M: Mg, Li, Cs and n: 1 or 2 depending of the cation type.

After the atomic basins are calculated for these systems, those can be integrated so as to get other properties for each atomic species. In Figure 6a, it depicts the atomic charges (*q*) for the N atom which are not different in magnitude nor in impact from the previous analysis in Section 2.3. All three cation cases lead to the increase in q, which is consistent at strong, near and mid-range interactions from the cations with N_2_.

In the case of the number of electrons (Figure 6b), these decrease as the cations start approaching to the molecule. As was interpreted before, most of these tend to be located at the triple bond, increasing the electron density at this region. However, due to the side-on configuration interaction of the cation with the N_2_ molecule, part of this density also gets polarized toward the cation. In this sense the delocalization index (DI) [46,47] property appears which can be related directly with the bond order denoting the number of electron pairs shared between two bonded atoms. Then, the DI is the magnitude of the exchange of the electrons in the basin of atom A with those in the basin of atom B(λ(*A*,*B*)). For a closed-shell system, this is defined as follows:

(2) $$\lambda (A,B)=2\left|F^{\alpha }(A,B)\right|+2\left|F^{\beta }(A,B)\right|,$$

where the Fermi correlation is defined as follows:

(3) $$\left|F^{\alpha }(A,B)\right|=-\sum_{i}\sum_{j}\int_{A}dr_{1}\int_{B}dr_{2}\left\{\phi_{i}^{*}\left(r_{1}\right)\phi_{j}\left(r_{1}\right)\phi_{j}^{*}\left(r_{2}\right)\phi_{i}\left(r_{2}\right)\right\}=-\sum_{i}\sum_{j}S_{ij}(A)S_{ij}(B)$$

where $S_{ij}$(A) = $S_{ij}$(B) is the overlap integral of two spin orbitals over a region Ω and S represents spin (*α* or *β*). Thus, in simple terms, the delocalization index λ(A,B) gives the number of shared or bonding electrons between atoms A and B. From Figure 6c, the DI decreases as well as the cation distance from the N_2_ molecule also does. This means the weakening of the triple bond. In the case of Cs^+^, these phenomena are more abrupt, which a priori is indicative of the pseudo-covalent interaction, as will be shown later.

#### 2.6.5. Localization and Delocalization Description

The delocalization index (DI) is also a continuous measure of the electronic comparison between two fuzzy atom regions [48]. For a free N_2_ molecule, DI = 3.11 is observed, showing the presence of three shared electron pairs into the N–N bond (Table 5). However, the presence of a cation makes the DI decrease. A DI > 3 indicates a shared electron overload, by a great delocalization or in this case a polarization induced by the metal center.

Alkali metals (Li–Cs) exhibit high DI values (~3.03–3.06), close to the reference value for free N_2_ (DI ≈ 3). This suggests that the electron delocalization in the N–N bond is similar to that of the non-activated triple bond. Mg^2+^ shows a reduced DI (2.83) and Ca^2+^ presents an intermediate value (2.94), where electrostatic coupling appears to predominate over direct electron density transfer.

The location index (LI) quantifies the total electron density localized in the nitrogen orbitals involved in their interaction [48]. In the free N_2_ molecule, six are distributed between the α and π orbitals that conform to the triple bond (LI = 5.44). Alkali metals (~5.36–5.39) reflect electron localization similar to the unperturbed system. Mg^2+^ exhibits the lowest LI (5.11) and Ca^2+^ shows a value of 5.27, indicating reduced electron localization on the nitrogen atoms, consistent with a more covalent interaction and greater electron redistribution induced by the cation’s field.

The intrinsic bond strength index (IBSI) evaluates the electron density at the bond critical point, where higher values characterize strong covalent bonds and lower values indicate weaker interactions [49]. For a free N_2_ molecule, IBSI is approximately 2.1, corresponding to a strong covalent bond. Alkali metals show similar IBSI values (~2.17–2.18), slightly lower than that of free N_2_. Mg^2+^ and Ca^2+^ induce a lower IBSI value with 2.13 and 2.16, respectively, reflecting moderate bond weakening. Despite the observed reductions, covalent interactions remain significant in all systems.

#### 2.6.6. Non-Covalent Interaction Plots

Non-covalent interactions (NCIs) comprise a fundamental set of intermolecular forces. These interactions including electrostatic attraction and repulsion forces, hydrogen bonding, van der Waals effects, and dispersion forces represent key mechanisms through which molecules recognize, organize, and influence each other within their molecular environment [50,51].

**Figure 7:**
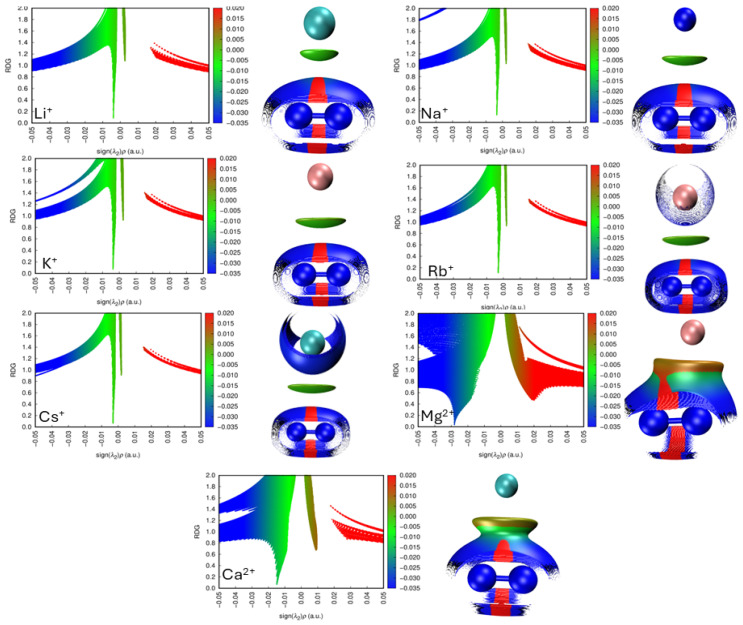
Analysis of Non-Covalent Interactions: Comparison between 2D Dispersion Maps and 3D NCI Representations in Complexes with Alkali and Alkaline-Earth Cations.

The presented scheme (Figure 7) establishes a classification of non-covalent interactions based on the parameters of *ρ* and the second eigenvalue of the electron density Hessian (λ_2_). At the extreme of strongly repulsive interactions (represented in red), significant steric effects are identified. In the intermediate region (green tones), van der Waals interactions are found, characterized by electron density values close to zero (*ρ* ≈ 0) and λ_2_ approximately null. The strongly attractive interactions (represented in blue) with positive electron density values (*ρ* > 0) and negative λ_2_ include hydrogen bonds, halogen bonds, and other directional interactions.

In the case of alkali metals, a predominance of van der Waals-type interactions is observed, manifested as induced dipoles between nitrogen and the metal, leading to relatively symmetric charge distributions. Due to the low polarizability of the lighter metals, the interactions tend to be weak and diffuse. In contrast, the heavier metals such as K, Rb, and Cs exhibit a slight increase in interaction strength as a result of their higher polarizability.

As the atomic size increases, the spatial distribution of the interaction regions around N_2_. It becomes more homogeneous and symmetric, whereas lighter metals exhibit more anisotropic patterns with differences in the upper and lower region near to the metal center.

In the alkaline-earth metal cases, a markedly different behavior is observed: A stronger inductive effect from the metal toward N_2_ is evident, reflected in the higher intensity of the visualized interactions and the prominent red (repulsive) regions in the plots. This electrostatic effect induces polarization within the nitrogen molecule, causing the N atoms to move slightly apart. Nevertheless, attractive regions (blue) between the metal and N_2_ are also detected. Mg^2+^, due to its higher charge and smaller ionic radius, generates regions of higher electron density and stronger interaction. Ca^2+^, being larger, displays more delocalized and moderately intense interactions.

## 3. Discussion

### 3.1. Comparison of Variables

**Figure A2:**
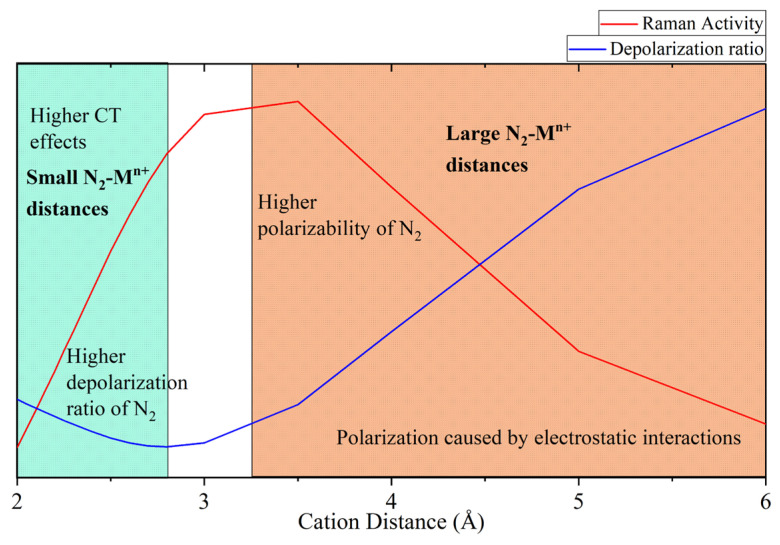
Trends of the cation interactions at small and large distances with the dinitrogen molecule.

In particular, the RA maxima and τ minima do not occur at the same N_2_–M^n+^ distance, suggesting different mechanisms for cation–dinitrogen interactions. At the RA maximum, cations induce stronger polarization of N_2_. However, the enhanced Raman signal simultaneously strengthens N–N covalence, thereby preserving the molecular symmetry. Since neither ionic nor covalent interactions, nor significant charge transfer (CT) between M^n+^ and N_2_, are evident at large N_2_–M^n+^ distances (i.e., RA maxima at 3–4 Å), the interaction at this regime can be described predominantly as electrostatic (Figure A2). In this case, the N_2_ electron cloud is perturbed according to the second interaction mode of cations (Figure 3d), leading to an equatorial compression of the molecule. The observed increase in the RA signal and the corresponding enhancement of N–N covalence can thus be attributed to the redistribution of electron density in the presence of the cation.

In contrast, ρ minima occur at shorter N_2_–M^n+^ distances, where RA values are small (Figure A2). This indicates that cations interact ionically or covalently with dinitrogen. Here, the proximity of the cation induces the first interaction mode (Figure 3d), increasing the polarizability of the N_2_ electron density along the equatorial plane. This effect is likely responsible for promoting N–N bond stretching. Consequently, larger reductions in MBO(N–N) are expected at shorter N_2_–M^n+^ distances, in agreement with previous observations.

Because N_2_ is a highly nonpolar, symmetric molecule, and the cation is equidistant from both nitrogen atoms, ideal polarization alone cannot significantly alter the molecular dipole moment. Thus, the cation’s effect on the N_2_ triple bond is symmetric. However, this idealized picture is modified by the μ and φ parameters specific to each cation, which define the mechanism by which they act on N_2_.

**Figure 8:**
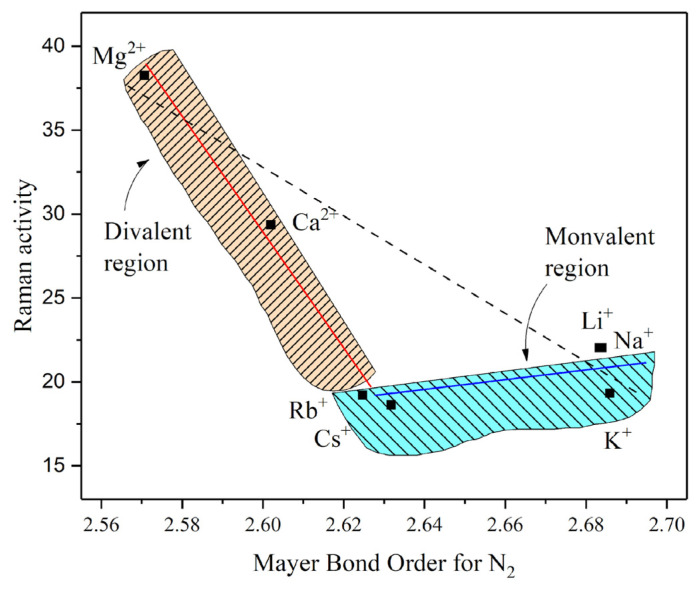
Distribution of AM cations comparing MBO(N–N) with Raman activity, using the M^n+^–N_2_ distance minimums found in the MBO analysis.

Figure 8 compares MBO(N–N) minima with RA(N_2_), showing two distinct regions: one for monovalent cations and another for divalent cations. In the divalent region, lower MBO(N–N) values are observed as the cation radius decreases or as ionic potential and polarizing power increase. Analysis of the MC(N_2_) curves reveals that Mg^2+^ and Ca^2+^ promote stronger CT to N_2_. Hence, electron transfer plays a central role in N_2_–M^2+^ interactions. A similar effect is also seen among monovalent cations but is largely restricted to Li^+^, Na^+^, and K^+^. Nonetheless, the impact is much more pronounced with divalent cations, where both CT and polarization contribute. The latter effect arises from higher μ and φ values, which enhance deformation of the N_2_ electron cloud and facilitate CT between cations and the molecule.

No clear trend emerges when monovalent cations are considered a group, suggesting little dependence on ionic radii or related parameters. However, examining them individually reveals distinct mechanisms for MBO(N–N) reduction. Li^+^, Na^+^, and K^+^ exhibit the largest μ and φ values within the monovalent series, as well as noticeable CT compared to Mg^2+^ or Ca^2+^. This interpretation is supported by the MC(N_2_) analysis. Yet, the gap separating these ions from the divalent species (Figure 2b) suggests that the μ and φ of Li^+^, Na^+^, and K^+^ remain insufficient to effectively polarize N_2_. As a result, their ability to interact with dinitrogen is constrained, consistent with the poor MBO(N–N) reductions observed (Figure 8). Thus, their interaction combines CT and polarization effects, though to a lesser extent than in divalent systems.

Rb^+^ and Cs^+^ represent special cases. Their comparatively low μ and φ values (Table 1) lead to much weaker effects on MBO(N–N) than those of lighter monovalent cations. For these ions, the interaction with N_2_ is primarily electrostatic and determined by the relative energy alignment of their molecular orbitals with those of dinitrogen.

### 3.2. Analysis of the Electron Cloud of Each N_2_-M^n+^ System

The N_2_ electron cloud is expected to undergo different deformations depending on the influence of the cationic species, which play a crucial role in determining the degree of activation of the molecule. To assess these deformations, electron localization function (ELF) plots were generated for each critical point identified in the N_2_–M^n+^ vs. MBO(N–N) analysis.

**Figure 9:**
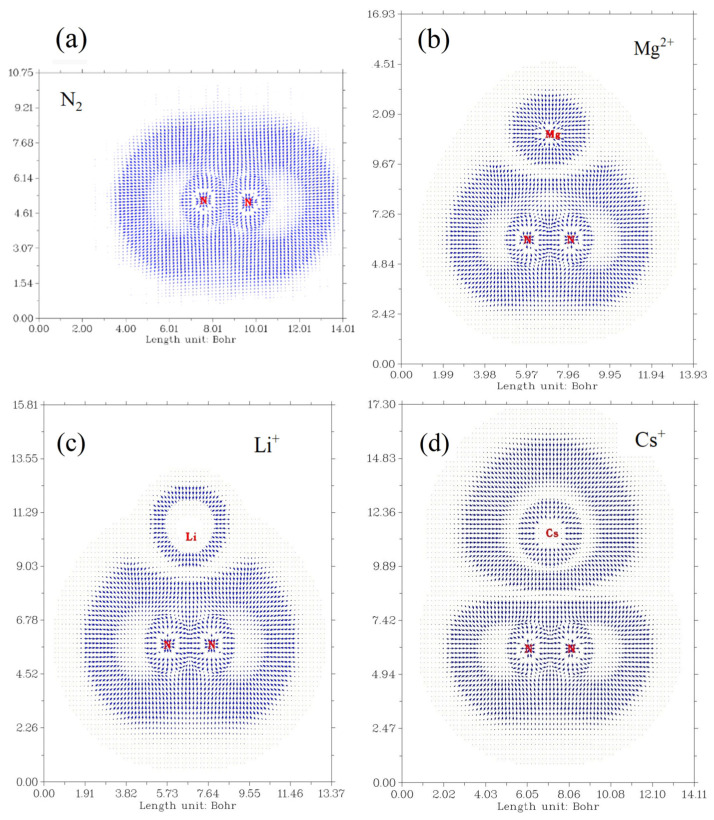
ELF plot of an (**a**) isolated dinitrogen molecule which is defined as the base system for polarization effects of cations. Representative AM cation effect in dinitrogen molecule using bidimensional ELF plots for, (**b**) N_2_–Mg^2+^ system, (**c**) N_2_–Li^+^ system, and (**d**) N_2_–Cs^+^ system.

**Figure 10:**
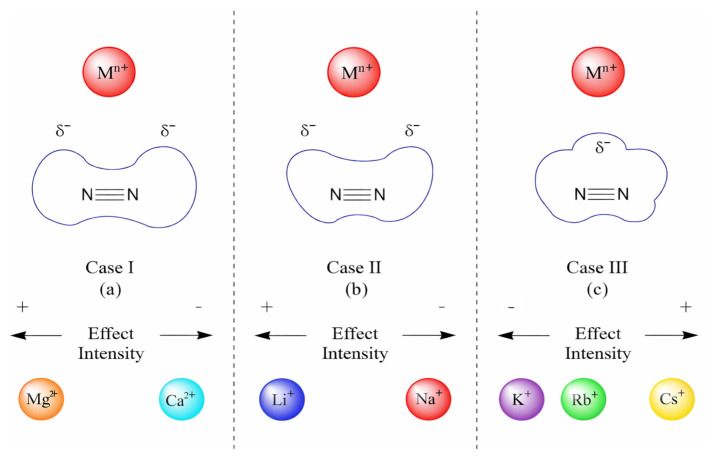
Ideal deformations for the electron cloud of dinitrogen caused by the effect of AMs species.

A comparison of the ELF plots in Figure 9a (i.e., bare dinitrogen) with those in Figure 9b–d reveals three distinct patterns of electron density deformation relative to the base system. A schematic representation of these patterns is provided in Figure 6. In the Mg^2+^ system (Figure 9b and Figure S2), two protuberances in the N_2_ electron density (ED(N_2_)) appear, slightly tilted toward the cation. At the same time, no noticeable deformation is observed in the Mg^2+^ electron density itself, suggesting that this cation possesses strong polarization power. A similar, though weaker, effect is seen for Ca^2+^ (Figure S3). This behavior can therefore be attributed to the combined effects of CT and polarizability exerted by these cations on dinitrogen (refer to case I in Figure 10).

A second case (refer to case II in Figure 10 and Figure S4) is exemplified by the Li^+^ system, where two protuberances of the ED(N_2_) are again observed. However, unlike in the Mg^2+^ case, these do not tilt directly toward the cation. In addition, the polarization of Li^+^ itself is minimal (Figure 9c), reflecting its limited ability to polarize N_2_. A similar pattern is observed for the N_2_–Na^+^ system (Figure S5).

In contrast, a third case (refer to case III in Figure 10 and Figures S6–S8) is observed for larger cations (Cs^+^, Rb^+^, K^+^), most clearly exemplified by Cs^+^ (Figure 9d). In this case, no side protuberances are present; instead, a single protuberance appears in the center of the ED(N_2_). Owing to the low CT values of these cations, which stem from their weak ionic activity, the reduction in MBO(N–N) appears to be the most plausible explanation for the modification of the electron cloud in the presence of Cs^+^ and Rb^+^. However, this analysis alone is insufficient to fully describe the electronic interactions in case III. To gain deeper insight, molecular orbital (MO) analysis is required to clarify the underlying mechanism.

### 3.3. Contrasting the ELF Results with a N_2_-M^+^ Molecular Orbital Analysis

Because of the highly ionic nature of alkali and alkaline-earth cations and the inertness of dinitrogen (a highly covalent system), covalent interactions between N_2_ and M^n+^ are unlikely. This hypothesis is largely supported for small cations, where the original MOs(N_2_) are preserved (Figure S9). In these cases, the main phenomenon is CT, and the perturbation of the orbitals arises from the polarization of N_2_ MOs by the nearby atomic orbitals (AOs) of the cation in the virtual region.

Covalent contributions from cations are negligible, as their electron density in the N_2_–M^n+^ MOs is below 5% and remains balanced for Cs^+^, Rb^+^, and K^+^ (Table 6). To account for CT effects, the empty AOs of the cation must be included in the binding MOs of the N_2_–M^n+^ system. This requires the presence of cationic AOs that differ from the ground-state electronic configuration. Table S1 lists the empty AOs contributing to the HOMO (H), H–1, H–2, H–3, and H–4 MOs of the N_2_–M^n+^ systems. Mg^2+^, Ca^2+^, Li^+^, and Na^+^ all exhibit empty AOs that contribute to binding MOs. The participation of occupied cation AOs increases in the order: K^+^ > Na^+^ > Li^+^ > Ca^2+^ > Mg^2+^, consistent with the relative strength of CT effects.

**Figure 11:**
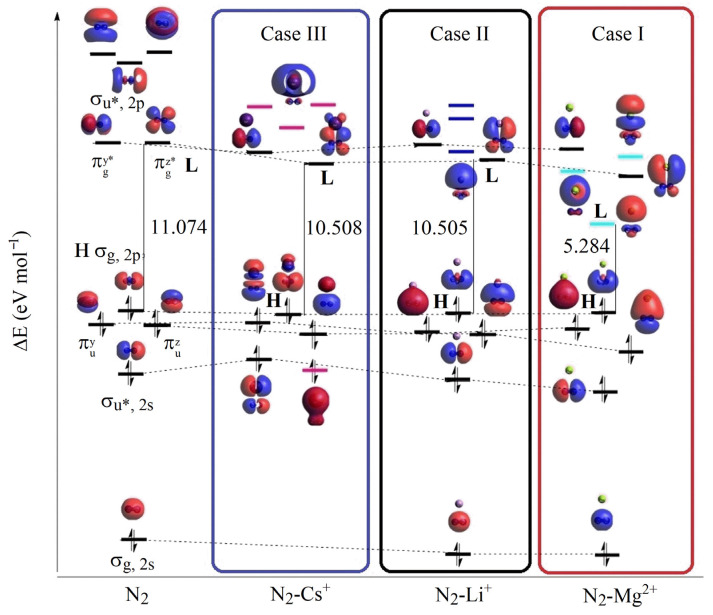
Molecular orbitals diagram for the main dinitrogen electron cloud polarizations. Case I (N_2_–M^2+^), Case II (N_2_–Li^+^), and Case III (N_2_–Cs^+^).

For Mg^2+^, the deformation of N_2_ electron density (case I, Figure 10) results from the overlap of cationic s- and p-type orbitals with the π* MOs of N_2_ (Figure 11, case I). This overlap polarizes the molecule along the molecular axis. The high μ and φ values perturb the N_2_ electron density, while the presence of unoccupied Mg^2+^ orbitals in the binding MOs allows them to accept polarized density. As a result, part of the total ED(N_2_) is drained, weakening the MBO(N–N). This is evident in the H, H–1, and H–2 MOs (Figure 11, case I; Table S7). The loss of polarized ED(N_2_) also maximizes the fraction of covalent ED(N_2_), which correlates with the strong RA signal.

A comparable phenomenon is observed in Ca^2+^. Here, the proximity of d-type orbitals to the LUMO (π* MOs of N_2_) induces polarization along the axis. This polarized density is absorbed into the unoccupied Ca^2+^ orbitals, particularly in the H, H–1, and H–2 MOs (Figure S9; Table S7). The CT effect is weaker than for Mg^2+^, owing to the smaller fraction of unoccupied orbitals contributing to binding. Nevertheless, the combined CT and polarizing effects remain significant. At a cation distance of 3 Å, MBO(N–N) reaches its minimum, consistent with maximal drain of polarized ED(N_2_) and maximal retention of covalent character, similar to Mg^2+^.

For Li^+^ (Figure 11, case II), only one s-type AO contributes to the π* MOs of N_2_, resulting in weaker polarization compared to divalent cations. The lower μ and φ values reduce its ability to perturb ED(N_2_) and attract electron density, thereby limiting CT effects. The main influence in case II is thus the polarization of ED(N_2_) by proximal AOs, with CT playing only a minor role.

Na^+^ behaves similarly to Li^+^. The perturbation of ED(N_2_) is dominated by polarizing effects of Na AOs on the π* MOs, while CT is inefficient. Although unoccupied Na orbitals participate in binding MOs, the simultaneous contribution of occupied Na orbitals diminishes net CT.

These monovalent cations are most effective at short distances (2.4–2.6 Å), where they polarize N_2_ along the equatorial plane (perpendicular to the bond axis). This drives electron density to the “corners” of the molecule, limiting CT. For Li^+^ and Na^+^, polarization by orbitals near the LUMO and HOMO is insufficient to strongly activate dinitrogen.

Cs^+^ provides a reference point for case III. Its large ionic radius yields very low μ and φ values, greatly reducing its ability to perturb ED(N_2_). Thus, Cs^+^ primarily interacts electrostatically, with minimal CT (Table S7). As a result, changes in MBO(N–N) are small, with negligible participation of the unoccupied AOs(Cs) in the binding of MOs(N_2_-Cs^+^). Nevertheless, the MO diagram of N_2_–Cs^+^ (Figure 11, case III) reveals a Cs AO near H–3 (pink highlight), which shifts HOMO levels upward (H–2) and downward (H–1). This perturbation produces a central protuberance in the N_2_ electron density, consistent with weak electrostatic effects. In the H–4 orbital, some electron density transfer from N to Cs produces a pseudo-covalent interaction. Overall, Cs weakly polarizes N_2_, while being slightly polarized itself by the molecule, resulting in mutual delocalization of electrons and a moderate reduction of MBO(N–N).

Rb^+^ behaves analogously but with slightly weaker effects than Cs^+^. In the MO diagram (Figure S9), an Rb AO near H–3 produces mild polarization and electron delocalization, along with pseudo-bonding. The slightly higher CT and polarizing power of Rb^+^ compared to Cs^+^ enhance its impact. In both Cs^+^ and Rb^+^, the reduction in MBO(N–N) is linked to the presence of cation AOs between the σ(2s) and σ*(2s) orbitals of N_2_, with stronger effects when closer to σ*(2s).

K^+^ exhibits the weakest activation of N_2_. It combines features of both light and heavy monovalent cations, effectively “canceling out” activation mechanisms. CT is limited because occupied AOs dominate the binding region, while polarization is inefficient in the virtual region. The position of occupied K^+^ AOs midway between σ(2s) and σ*(2s) is suboptimal (Figure S9), preventing pseudo-bonding.

Overall, the most effective activation occurs at N_2_–M^n+^ distances of 2.7–3 Å. At this range, strong polarization of the virtual region (particularly π* MOs) promotes CT effects, which weaken the N–N bond. Cations with sufficient ionic polarizability further promote pseudo-bonding and delocalization, amplifying activation effects.

### 3.4. Comparison with Existing Complexes

Although this work focuses exclusively on the side-on position of the cation, numerous experimental efforts have explored complexes with alkali metal ions which can adopt either side-on or end-on coordination modes. In multi-metallic complexes, it is clear that the main coordination geometry is dictated by the position of the transition metals but the neighboring alkaline control strategy allows us to modulate the degree of N_2_ activation.

Alkali ions are present in end-on mode only in mono-TM complexes or multi-TM complexes occupying the side-on position, leaving free the end-on position for the alkali. In an early study, Krüger et al. reported a (C_6_H_5_Li)_6_Ni_2_N_2_{(C_2_H_5_)_2_O}_2_ complex, where Ni are bound to nitrogen N_2_ in side-on η^2^ fashion and Li^+^ in end-on position. According to the authors, the six lithium cations organize into unique supramolecular architectures in which the N_2_ ligand coordinates perpendicularly to the Ni–Ni axis, forming a (–Li–Ni–N–)_2_ ring [52]. This arrangement enables elongation of the N–N bond through a synergistic σ-acceptor/π-donor interaction, where Li^+^ acts as a σ-acceptor and Ni as a π-donor. Overall, experimental evidence suggests that N–Li interactions preferentially adopt an end-on coordination geometry (1.96–2.09 Å) [27,53], whereas N–Na (2.19–2.48 Å) and N–K (2.53–2.72 Å) [27] show more variable tendencies toward this geometry.

End-on coordination of an alkali cation enhances the nucleophilicity of the N atom by electrostatically drawing electron density toward the terminal nitrogen of the N_2_ ligand. This induces a direct electronic effect that facilitates metal-to-ligand backbonding into the N_2_ π* orbitals. From a molecular orbital perspective, this effect arises from (i) polarization of orbital density and (ii) shifts in orbital energies caused by the nearby positive charge [27]. Hence, the cation’s proximity increases the electronegativity of the distal nitrogen, polarizing the N_2_ π (HOMO) toward the cation-bound atom and the N_2_ π* (LUMO) toward the metal-bound atom, so these can be considered electronic promoters. However, end-on coordination of alkali cations to N_2_ ligands often requires their encapsulation by coordinating solvents or crown ethers [29,53] which eliminates most of the time the direct formation of multimetallic complexes.

Comparison of the calculated N_2_–M^n+^ distances in this report (Table S8) with literature values shows good agreement with experimental data for side-on coordinated species such as N–Na (2.28–2.97 Å), and N–K (2.70–2.88 Å) [27,38,54,55]. In related Ti–K complexes reported by Doyle et al., the potassium cations in [((CH_2_CH_2_NSiMe_3_)_3_))Ti]_2_(μ-η^1^:η^1^:η^2^:η^2^-N_2_)K_2_ act as electrostatic bridges that stabilize the negative charge accumulated on the N_2_ ligand [56]. This electrostatic stabilization cooperatively shortens the Ti–N_2_ distance and elongates the N–N bond, characteristic of side-on coordination. Similar structural motifs are observed in systems such as (μ-1,2-dinitrogen)bis(tris(neopentyl)vanadium) derivatives containing Na^+^ and K^+^ ions, [[K(THF)_2_]-[K_2_(μ-THF)]{([O_3_C]Ti)_2_(μ-1,2-N_2_)}]^−^, K_2_{[(${tren}^{TMS}$)Ti]_2_(μ-1,2-N_2_)}, and [K(THF)]_2_[(Mo_3_TiS_4_)_2_(μ-1,2-N_2_)]. Analogous coordination patterns are also found in bis(β-diketiminato)cobalt(μ-1,2-dinitrogen) complexes and their Co/Ni congeners [24], highlighting the structural diversity and rich coordination chemistry characteristic of μ-1,2-dinitrogen bridged system.

In general, side-on coordination allows cations to stabilize multimetallic frameworks, where arene-type ligands, for instance, promote cation–π interactions that expand the metal core and prevent transition-metal centers from approaching too closely. In the Fe–M systems described by Holland et al., the size of the alkali cation (K^+^, Rb^+^, or Cs^+^) governs the nuclearity of the cluster (Fe_4_ vs. Fe_3_) and determines whether the N_2_ molecule undergoes full cleavage to nitrides or only partial activation in triangular Fe_3_(μ-N_2_)_3_ structures [57]. Cation-directed supramolecular architectures create specific electronic and steric microenvironments that enable the cooperative approach of multiple metal centers to N_2_. These architectures facilitate activation through an electrostatic “push–pull” mechanism, in which the cation is not a passive spectator but an essential component of the active site. In the same line of systems, attempts to synthesize Li_2_[^Me^LFe]_2_(μ-N_2_) have been unsuccessful, likely because the small ionic radius of Li^+^ prevents it from effectively occupying the arene cavity, leading to decomposition [27]. However, such complexes may still be stabilized by alternative ligand environments.

In such a way, side-on coordination enables cooperative N_2_ activation involving both metal centers and cations, supporting the view that multimetallic mechanisms are essential in N_2_ cleavage—similar to the rate-limiting step in industrial ammonia synthesis, which proceeds via transition states spanning multiple Fe sites. Notably, several rare-earth complexes also exhibit a preference for side-on coordination, resulting in multimetallic assemblies whose catalytic or chemical activity can be further augmented through the inclusion of alkali metal cations [24].

From the full picture, alkali cations with an end-on coordination to N_2_ ligands are typically more distant from the TM center, unlike in the classical Mittasch Fe–O–K catalyst [58], where potassium exists as an Fe-O-K unit. Nonetheless, the presence of a nearby positive charge with a side-on coordination enhances metal-to-N_2_ backbonding, increasing electron density near the cation and stabilizing key intermediates during multi-step N_2_ reduction.

### 3.5. Defining 3 Different Cases

Over the years, there has been growing interest in the study of covalent interactions between transition metals (TMs) and dinitrogen. However, the true role of non-covalent interactions with N_2_ remains poorly understood.

Overall, the results demonstrate that the MBO (N–N) decreases systematically as cations approach the N_2_ molecule which agrees with the chemical properties of the cationic species. In such a way, best models will be those with small ionic radius and high ionic potential. Cations with high ionic potential also exhibit strong polarizing power, effectively acting as concentrated charge packets. Consequently, their ability to distort the electron density of N_2_ is enhanced, particularly at short N_2_–M^n+^ distances where electron transfer plays a central role.

As a preliminary exploration of this issue, our findings indicate that non-covalent interactions play a crucial role in the activation of neutral N_2_ molecules. Computational analysis of alkali and alkaline-earth metal (AM) cations revealed multiple interaction mechanisms that depend on the nature of the cationic species. Collectively, these cations polarize N_2_, lowering the energy of the π*(N_2_) orbitals and thereby inducing a pre-activation state that facilitates subsequent interactions with metalorganic structures involving TMs, acting as auxiliary components for the main catalyst.

This study identified three main interaction domains among IA and IIA cations. The first domain, Case I, encompasses alkaline-earth cations (Mg^2+^ and Ca^2+^). These species strongly polarize N_2_, inducing charge-transfer (CT) effects in which polarized electron density from N_2_ is transferred into the unoccupied orbitals of the cation. The resulting weakening of the N≡N triple bond is reflected in a reduced bond order and a slight bond elongation. These effects are attributed to the high ionic potential and polarizing power of Mg^2+^ and Ca^2+^, which enable them to engage in substantial interactions with N_2_.

The second domain, Case II, includes the lighter alkali cations (Li^+^ and Na^+^). These ions also exert polarization effects, but their energy mismatch with the π*(N_2_) orbitals results in less efficient polarization and reduced CT contributions compared to Case I.

The third domain, Case III, is represented by the larger alkali cations (K^+^, Rb^+^, and Cs^+^). Here, polarization is governed not by ionic potential or polarizing power, but primarily by electrostatic interactions. Due to their large radii, these cations are easily polarizable, which allows them to delocalize the electron density of N_2_ upon polarization. This phenomenon leads to the formation of pseudo-bonding interactions between the cation and the nitrogen atoms.

Overall, among the three domains, divalent cations emerge as the most effective for pre-activating N_2_. However, the monovalent cations can induce a difference in the shape of the electronic cloud which could result in better synergy with transition metal classical activation. The choice of cation must still be tailored to the chemical environment, geometry, reactivity, and structure of the host metalorganic complex. Additionally, this work establishes optimal cation–N_2_ distances for maximum interaction: 2.4–2.6 Å for light alkali cations, 2.7–3.0 Å for alkaline-earth cations, and 2.8–3.0 Å for heavier alkali cations. Together, these insights provide a theoretical framework to guide the experimental use of cationic species in N_2_ activation strategies.

## 4. Materials and Methods

**Figure 12:**
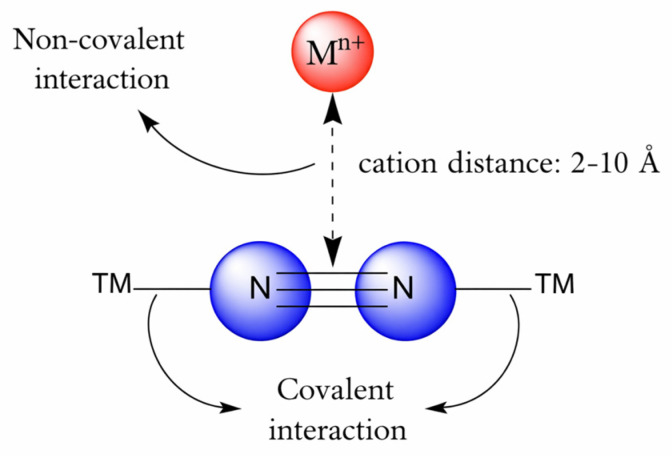
M^n+^-N_2_ side-on interaction system. With M: Li^+^, Na^+^, K^+^, Rb^+^, Cs^+^, Mg^2+^, and Ca^2+^.

The initial structures of molecular nitrogen and dinitrogen–cation (N_2_–M^n+^) systems were constructed using Avogadro 1.2.0 software [59], considering a side-on interaction mode for the latter (Figure 12). Pre-optimizations were performed with the Universal Force Field (UFF) in the Avogadro package. Density functional theory (DFT) calculations were then used to further optimize these systems in Cartesian coordinates with ORCA 4.2.1 [60]. The N_2_–M^n+^ sets (M = Li^+^, Na^+^, K^+^, Rb^+^, Cs^+^, Mg^2+^, Ca^2+^) were optimized using the B3LYP functional [61], the def2-QZVP basis set, and D3BJ dispersion corrections [62,63]. To establish side-on interactions between cations and N_2_, several constraints were applied to fix the cation positions, which were systematically varied from 2 to 10 Å for each case [38].

Once optimized, FTIR and Raman spectra were calculated to evaluate the polarization effects of the cations on dinitrogen. The results achieved with the chosen level of theory were contrasted with CAM-B3LYP [64], M06-2X [65] and CSSDD(T) [66,67] methodologies to verify their significance.

Population analysis data were obtained using Mulliken [68], Löwdin [69] and Atomic Dipole Moment Corrected Hirshfeld (ADCH) analysis [70,71]. Mayer [72,73] and Löwdin (LBO) Bond Orders were calculated from ORCA, while Fuzzy [48] (FBO) and Laplacian [74] (LBO) Bond Orders were computed by the Multiwfn package [75]. Interaction energies were determined from enthalpy differences.

Intrinsic Bond Strength Index (IBSI) was calculated using the IGMI model with a high-quality integration grid [49]. Furthermore, the Localization (LI) and Delocalization (DI) Index [48,76] was based on Becke’s partitioning of molecular space into atomic regions (QTAIM) which were analyzed by using Multiwfn and AIMII software [77,78] as well as to get other miscellaneous properties from the integration of the atomic basins. Finally, the study of Non-Covalent Interactions (NCI) was obtained through Reduced Density Gradient (RDG) analysis implemented in Multiwfn [50,51]. Two-dimensional scatter plots and three-dimensional representations (RDG vs. sign(λ_2_)*ρ*) were generated, and the color scheme allows for visual distinction between attractive interactions (blue shades), van der Waals interactions (green), and repulsive interactions (red), colored according to sign(λ_2_)*ρ* values.

To complement these results, electron localization function (ELF) analyses [79,80] were performed with the Multiwfn package. To gain deeper insights, molecular orbital theory (MOT) was employed to interpret the behavior of the different AM species. All MOT analyses were conducted on the previously optimized systems. Together, these approaches enabled classification of the cationic systems and identification of distinct behaviors among alkali and alkaline-earth cations.

## 5. Conclusions

Whether for N_2_ or CO_2_ activation, we observed a recent trend in organometallic catalysis where not only transition metals are used but also alkali or alkaline-earth cations are present in the vicinity of the molecule desired to be activated. In this work, we focused on this peculiar cation effect with the intent of rationalizing the phenomenon through different descriptors.

While the bond order or charge descriptors allow us to partially capture the nature of the effect, single-numbered descriptors are insufficient to capture more subtle changes. The plot of ELF and the orbitals have been particularly decisive in obtaining deeper insights. The charge and polarizability of the cation is evidently important, but there is also deformation of the electronic cloud which can adopt different shapes. The alkali and alkaline-earth cations can act as catalysts through Non-Covalent Interactions which can decrease the electron density in the center of the N_2_ bond or induce deformation on the side of nitrogen. This effect is highly dependent on the nature of the cations, and based on orbital energy levels, we can define 3 different cases. Case I, consisting of divalent cations, seems to be the most efficient in decreasing the electron density in the center of the bond through weak charge transfer and thus helps with the activation of N_2_. On the other side, case III cations (Cs, Rb. K) involve an increase in the center of the bond, increasing the covalent contribution in N_2_. Case II cations display non-symmetric deformation of the electronic cloud. The 3 cases we define are particularly important, as they define different physical aspects which can have a different synergy with transition metals. While case I cations would be more efficient alone, case II cations induce deformation of the electron, resulting in greater asymmetry, and case III could allow for a slight change in the covalent/ionic degree of the N_2_ bond.

To explain the synergy effect occurring in future complexes, we recommend not only looking at classical descriptors but also at the electronic density and the orbitals. Beyond rationalizing the behavior, this information can be used in further development of new efficient complexes including both transition metals and alkali or alkaline-earth cations, where cations representative from each case would bring a different contribution.

## Figures and Tables

**Table 1:** Chemical parameters for alkali and alkaline-earth cations.

Cation	Ionic radii 6-C ^a^ (Å)	μ 6-C	ϕ 6-C	α (Å^3^)
Li	0.76	1.316	1.731	24.33 [43]
Na	1.02	0.980	0.961	24.11 [44]
K	1.38	0.725	0.525	43.4 [43]
Rb	1.52	0.658	0.433	47.24 [45]
Cs	1.67	0.599	0.359	59.42 [45]
Mg	0.72	2.778	3.858	10.6 [43]
Ca	1	2	2	22.8 [43]
Cation	Ionic radii 8-C ^b^ (Å)	μ 8-C	ϕ 8-C	
Li	0.92	1.087	1.181	-
Na	1.18	0.847	0.718	-
K	1.51	0.662	0.438	-
Rb	1.61	0.621	0.386	-
Cs	1.74	0.575	0.330	-
Mg	0.89	2.247	2.525	-
Ca	1.12	1.786	1.594	-

^a^ 6-C: coordination number six. ^b^ 8-C: coordination number eight.

**Table 2:** Interaction energies of cation with N_2_ at optimized distance. The last column describes the bond dissociation energy of N_2_.

Cation	ΔH_M+n–N2_ (kJ/mol)	ΔH_N–N_ (kJ/mol)
Li	−23.80	921.55
Na	−18.24	927.11
K	−14.83	930.53
Rb	−14.15	931.21
Cs	−11.80	933.56
Mg	−118.99	826.36
Ca	−60.36	884.99
N–N _Ref_		945.35

**Table 3:** Atomic Charge Distribution in Nitrogen and Cation for Complexes with Alkali and Alkaline-Earth Metals.

Cation	Atomic Charges for N		
	Mulliken	Löwdin	Hirshfeld
Li	0.057	0.174	0.037
Na	0.041	0.121	0.026
K	0.023	0.082	0.045
Rb	0.020	0.070	0.038
Cs	0.017	0.064	0.035
Mg	0.087	0.147	0.086
Ca	0.077	0.162	0.075

**Table 4:** N–N Bond Orders in N_2_–M^n+^ Complexes.

Cation	Bond Order N–N			
	Mayer	Löwdin/Wiberg	Laplacian	Fuzzy
N_2 Ref_	2.865	3.939	3.2225	3.115
Li	2.690	3.711	3.211	3.062
Na	2.704	3.785	3.212	3.031
K	2.710	3.841	3.215	3.049
Rb	2.707	3.859	3.215	3.035
Cs	2.722	3.867	3.219	3.046
Mg	2.599	3.523	3.124	2.833
Ca	2.614	3.725	3.186	2.937

**Table 5:** Electron Delocalization, Localization, and Bond Strength Indices for N–N Interactions in Alkali and Alkaline-Earth Cation Complexes.

Cation	Delocalization Index N–N	Localization Index of N	Intrinsic Bonds Strong Index N–N
N_2ref_	3.115	5.442	2.181
Li	3.062	5.393	2.176
Na	3.031	5.362	2.177
K	3.049	5.379	2.180
Rb	3.035	5.364	2.180
Cs	3.046	5.374	2.182
Mg	2.832	5.1130	2.13
Ca	2.937	5.267	2.164

**Table 6:** Cation contribution to the bonding MOs of the system N_2_–M^n+^.

Cation	H ^a^ (%)	H ^a^–1 (%)	H ^a^–2 (%)	H ^a^–3 (%)	H ^a^–4 (%)
Li	2.59	0.77	3.67	0.79	0.18
Na	2.35	0.57	2.91	0.56	0.32
K	2.18	0.45	3.30	0.58	99.8
Rb	1.93	0.34	4.02	0.77	99.9
Cs	4.59	21.9	1.96	0.94	98.3
Mg	1.90	0.83	7.28	0.75	0.16
Ca	1.50	0.40	4.28	0.30	0.07

^a^ H: HOMO.

## Data Availability

The original contributions presented in this study are included in the article/Supplementary Materials. Further inquiries can be directed to the corresponding author, Thibault Terencio (tthibault@yachaytech.edu.ec).

## Supplementary Materials

The following supporting information can be downloaded at: https://www.mdpi.com/article/10.3390/ijms27031311/s1.

## Abbreviations

The following abbreviations are used in this manuscript:

DFT	Density Functional Theory
HOMO	Highest Occupied Molecular Orbital
LUMO	Lowest Unoccupied Molecular Orbital
H-B	Haber–Bosch
T-M	Transition Metal
IEs	Ionization Energies
AMs	Alkali and alkaline-earth Metals
MBO	Mayer Bond Order
MC	Mulliken Charges
RA	Raman Activity
ED	Electron Density
ELF	Electron Localization Function
MOs	Molecular Orbitals
AOs	Atomic Orbitals
CT	Charge Transfer
QTAIM	Quantum Theory of Atoms in Molecules
