# Supplementary material for: Neighboring Alkali Cations as an Efficient Strategy for N2 Activation: A DFT Analysis
Source: Int J Mol Sci. 2026 Jan 28;27(3):1311. doi: 10.3390/ijms27031311 (PMC12898064; doi:10.3390/ijms27031311)
Supplement: Supplementary file 1 [file ijms-27-01311-s001.zip › ijms-3914170-supplementary.pdf]

Neighboring Alkali Cations as One Efficient Strategy for N_2 Activation: A DFT Analysis

Jean C. Villa-Arpi[1,2]*, R. Guanuna[2,3], Juan P. Saucedo-Vazquez[2,3] and Thibault Terencio[2,3]**

[1] Departament de Ciència de Materials i Química Física Institut de Química Teòrica i Computacional, Universitat de Barcelona, c/ Martí i Franquès 1-11, 08028 Barcelona, Spain; jvillaar32@alumnes.ub.edu

[2] School of Chemistry and Engineering, Yachay Tech, 100119 Urcuqui, Ecuador; tthibault@yachaytech.edu.ec

[3] CATS INVESTIGATION GROUP (CAtalysis Theory & Spectroscopy), Yachay Tech, 100119 Urcuqui, Ecuador; tthibault@yachaytech.edu.ec

Index of contents

- QTAIM analysis for N_2-Mg^{2+}, N_2-Li^+, N_2-Cs^+ systems
    - Figure S1. Variation of the (3, –1) BCP N_2–N_1 Laplacian (∇^2_{BCP}) for N_2-Mg^{2+}, N_2-Li^+, and N_2-Cs^+ systems at different distance ranges. The bond critical points (BCP) are shown in green between the atomic species, while the N_2 – cation side-on configuration distance is shown implicit for each case ……..2
    - Table S1. Critical points (CP) properties for the *N_2-Mg^{2+}* system at 2.5. 2.7, 3.0 and 4.0 Å...3
    - Table S2. Properties from the integration of the atomic basins for the *N_2-Mg^{2+}* system, at 2.5, 2.7, 3.0 and 4.0 Å……..3
    - Table S3. Critical points (CP) properties for the *N_2-Li^+* system at 2.5. 2.7, 3.0 and 4.0 Å….. 4
    - Table S4. Properties from the integration of the atomic basins for the *N_2-Li^+* system, at 2.5, 2.7, 3.0 and 4.0 Å……..4
    - Table S5. Critical points (CP) properties for the *N_2-Cs^+* system at 2.5. 2.7, 3.0 and 4.0 Å…..5
    - Table S6. Properties from the integration of the atomic basins for the *N_2-Cs^+* system, at 2.5, 2.7, 3.0 and 4.0 Å……..5
- ELF analysis for each cation species
    - Figure S2. Effect of magnesium cation on the electron density of N_2 using ELF analysis…6
    - Figure S3. Effect of calcium cation on the electron density of N_2 using ELF analysis……..6
    - Figure S4. Effect of lithium cation on the electron density of N_2 using ELF analysis……..6
    - Figure S5. Effect of sodium cation on the electron density of N_2 using ELF analysis……..7
    - Figure S6. Effect of potassium cation on the electron density of N_2 using ELF analysis…..7
    - Figure S7. Effect of rubidium cation on the electron density of N_2 using ELF analysis…...7
    - Figure S8. Effect of cesium cation on the electron density of N_2 using ELF analysis………8
- MOs of the systems N_2-M^{n+}
    - Figure S9. Overall MOs diagram for each cationic species. Case I represented by magnesium cation, Case II represented by lithium cation and Case III represented by cesium cation ……...9
    - Table S7. Non-occupied Cation AOs contribution to Bonding MOs of the system N_2-M^{n+}...10
- Optimal performance of cations for activating N_2
    - Table S8. N_2–M^{n+} system interactions at which the cations exercise their maximum activity for each case in Side-On type coordination……..10

QTAIM analysis for N_2-Mg^{2+}, N_2-Li^+, N_2-Cs^+ systems

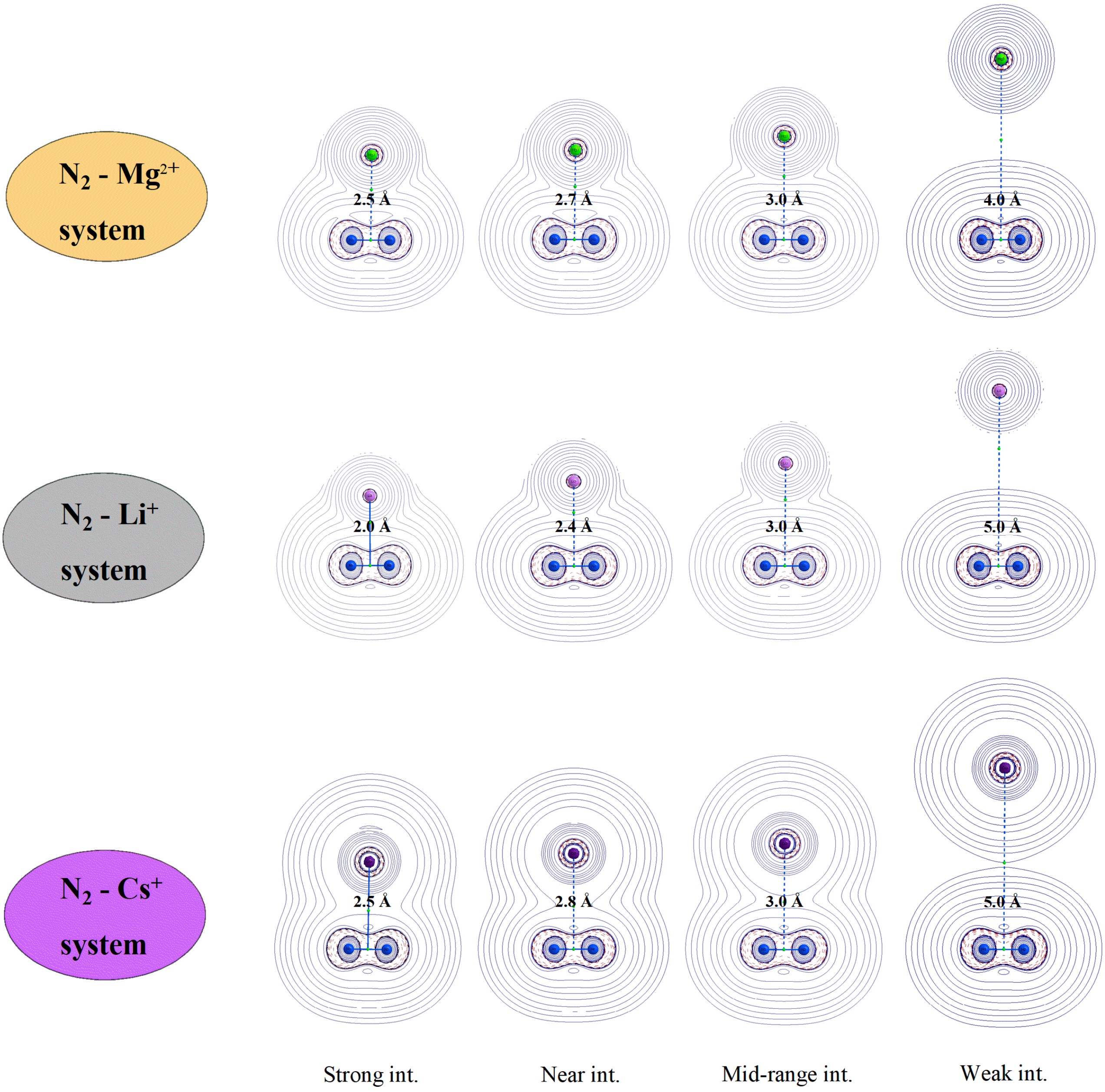

Figure S1. Variation of the (3, –1) BCP N_2–N_1 Laplacian (∇^2_{BCP}) for N_2-Mg^{2+}, N_2-Li^+, and N_2-Cs^+ systems at different distance ranges. The bond critical points (BCP) are shown in green between the atomic species, while the N_2 – cation side-on configuration distance is shown implicit for each case.

Table S1. Critical points (CP) properties for the N_2-Mg^{2+} system at 2.5. 2.7, 3.0 and 4.0 Å

N_2-Mg^{2+} distance (Å)	CP	Type	ρ $(electrons/{a_0}^3)$	$\rho_{nucleus}$ $(electrons/{a_0}^3)$	$\nabla^2{}_{BCP}$ $(electrons/{a_0}^5)$	ϵ	Bond radius GBL (a_0)
2.5	**CP1**	(3,-3) NACP N1	2.04E+02	2.04E+02	-	NA	-
	**CP2**	(3,-3) NACP N2	2.04E+02	2.04E+02	-	NA	-
	**CP3**	(3,-3) NACP Mg3	1.08E+03	1.08E+03	-	NA	-
	**CP4**	(3,-1) BCP N2 N1	7.31E-01	-	-3.13E+00	4.95E-02	2.06E+00
	**CP5**	(3,-1) BCP Mg3 N2	1.68E-02	-	6.60E-02	5.42E-01	4.72E+00
2.7	**CP1**	(3,-3) NACP N1	2.038E+02	2.038E+02	-	NA	-
	**CP2**	(3,-3) NACP N2	2.038E+02	2.038E+02	-	NA	-
	**CP3**	(3,-3) NACP Mg3	1.084E+03	1.084E+03	-	NA	-
	**CP4**	(3,-1) BCP N2 N1	7.307E-01	-	-3.119E+00	4.797E-02	2.062E+00
	**CP5**	(3,-1) BCP Mg3 N2	1.128E-02	-	3.710E-02	4.297E-01	5.097E+00
3.0	**CP1**	(3,-3) NACP N1	2.038E+02	2.038E+02	-	NA	-
	**CP2**	(3,-3) NACP N2	2.038E+02	2.038E+02	-	NA	-
	**CP3**	(3,-3) NACP Mg3	1.084E+03	1.084E+03	-	NA	-
	**CP4**	(3,-1) BCP N2 N1	7.292E-01	-	-3.096E+00	4.174E-02	2.062E+00
	**CP5**	(3,-1) BCP Mg3 N2	6.281E-03	-	1.525E-02	3.256E-01	5.663E+00
4.0	**CP1**	(3,-3) NACP N1	2.037E+02	2.037E+02	-	NA	-
	**CP2**	(3,-3) NACP N2	2.037E+02	2.037E+02	-	NA	-
	**CP3**	(3,-3) NACP Mg3	1.084E+03	1.084E+03	-	NA	-
	**CP4**	(3,-1) BCP N2 N1	7.241E-01	-	-3.013E+00	1.696E-02	2.062E+00
	**CP5**	(3,-1) BCP Mg3 N2	9.219E-04	-	2.858E-04	2.355E-01	7.553E+00

Table S2. Properties from the integration of the atomic basins for the N_2-Mg^{2+} system, at 2.5, 2.7, 3.0 and 4.0 Å

N_2-Mg^{2+} distance (Å)	Atom A	q(A)	L	K	K_{scaled}	μ (intraatomic)	G	N(A)	LI	DI	V_{ne}(A,A)
2.5	**N1**	3.487E-02	-5.457E-05	5.454E+01	5.454E+01	6.499E-01	5.454E+01	6.965E+00	5.427E+00	1.538E+00	-1.295E+02
	**N2**	3.489E-02	-3.631E-05	5.454E+01	5.454E+01	6.499E-01	5.454E+01	6.965E+00	5.427E+00	1.539E+00	-1.295E+02
	**Mg3**	1.930E+00	2.396E-06	1.984E+02	1.984E+02	4.795E-02	1.984E+02	1.007E+01	9.993E+00	7.672E-02	-4.698E+02
2.7	**N1**	3.058E-02	3.019E-06	5.454E+01	5.454E+01	6.491E-01	5.454E+01	6.969E+00	5.433E+00	1.537E+00	-1.295E+02
	**N2**	3.063E-02	2.521E-05	5.454E+01	5.454E+01	6.491E-01	5.454E+01	6.969E+00	5.433E+00	1.537E+00	-1.295E+02
	**Mg3**	1.939E+00	3.938E-06	1.984E+02	1.984E+02	4.591E-02	1.984E+02	1.006E+01	9.996E+00	6.507E-02	-4.697E+02
3.0	**N1**	2.552E-02	1.139E-05	5.454E+01	5.454E+01	6.434E-01	5.454E+01	6.974E+00	5.440E+00	1.535E+00	-1.664E+02
	**N2**	2.546E-02	-1.472E-05	5.454E+01	5.454E+01	6.434E-01	5.454E+01	6.975E+00	5.440E+00	1.535E+00	-1.664E+02
	**Mg3**	1.949E+00	6.162E-08	1.984E+02	1.984E+02	4.209E-02	1.984E+02	1.005E+01	9.999E+00	5.243E-02	-4.940E+02
4.0	**N1**	2.173E-02	3.957E-06	5.455E+01	5.455E+01	6.151E-01	5.455E+01	6.978E+00	5.442E+00	1.536E+00	-1.628E+02
	**N2**	2.174E-02	-3.399E-06	5.455E+01	5.455E+01	6.151E-01	5.455E+01	6.978E+00	5.442E+00	1.536E+00	-1.628E+02
	**Mg3**	1.957E+00	-4.993E-08	1.984E+02	1.984E+02	3.125E-02	1.984E+02	1.004E+01	1.000E+01	4.266E-02	-4.879E+02

Table S3. Critical points (CP) properties for the N_2-Li^+ system at 2.0. 2.4, 3.0 and 5.0 Å

N_2-Li^+ distance (Å)	CP	Type	ρ ($electrons/a_0{}^3$)	$\rho_{nucleus}$ ($electrons/a_0{}^3$)	$\nabla^2{}_{BCP}$ ($electrons/a_0{}^5$)	ϵ	Bond radius GBL (a_0)
2.0	**CP1**	(3,-3) NACP N1	2.037E+02	2.037E+02	-	NA	-
	**CP2**	(3,-3) NACP N2	2.037E+02	2.037E+02	-	NA	-
	**CP3**	(3,-3) NACP Li3	1.369E+01	1.369E+01	-	NA	-
	**CP4**	(3,-1) BCP N2 N1	7.279E-01	-	-3.045E+00	1.259E-02	2.062E+00
	**CP5**	(3,-1) BCP Li3 N2	2.555E-02	-	1.634E-01	6.009E-01	3.782E+00
**2.4**	CP1	(3,-3) NACP N1	2.037E+02	2.037E+02	-	NA	-
	CP2	(3,-3) NACP N2	2.037E+02	2.037E+02	-	NA	-
	CP3	(3,-3) NACP Li3	1.367E+01	1.367E+01	-	NA	-
	CP4	(3,-1) BCP N2 N1	7.279E-01	-	-3.055E+00	1.807E-02	2.062E+00
	CP5	(3,-1) BCP Li3 N2	9.603E-03	-	5.276E-02	4.129E-01	4.536E+00
**3.0**	CP1	(3,-3) NACP N1	2.037E+02	2.037E+02	-	NA	-
	CP2	(3,-3) NACP N2	2.037E+02	2.037E+02	-	NA	-
	CP3	(3,-3) NACP Li3	1.366E+01	1.366E+01	-	NA	-
	CP4	(3,-1) BCP N2 N1	7.267E-01	-	-3.039E+00	1.279E-02	2.062E+00
	CP5	(3,-1) BCP Li3 N2	2.118E-03	-	1.051E-02	2.751E-01	5.668E+00
**5.0**	CP1	(3,-3) NACP N1	2.037E+02	2.037E+02	-	NA	-
	CP2	(3,-3) NACP N2	2.037E+02	2.037E+02	-	NA	-
	CP3	(3,-3) NACP Li3	1.366E+01	1.366E+01	-	NA	-
	CP4	(3,-1) BCP N2 N1	7.253E-01	-	-3.013E+00	2.363E-03	2.062E+00
	CP5	(3,-1) BCP Li3 N2	1.123E-05	-	5.899E-05	1.096E-01	9.448E+00

Table S4. Properties from the integration of the atomic basins for the N_2-Li^+ system at 2.0, 2.4, 3.0 and 5.0 Å

N_2-Li^+ distance (Å)	Atom A	q(A)	L	K	K_{scaled}	μ (intraatomic)	G	N(A)	LI	DI	V_{ne}(A,A)
2.0	N1	1.679E-02	-8.807E-05	5.458E+01	5.458E+01	5.986E-01	5.458E+01	6.983E+00	5.450E+00	1.533E+00	-1.574E+02
	N2	1.683E-02	-1.150E-05	5.458E+01	5.458E+01	5.986E-01	5.458E+01	6.983E+00	5.450E+00	1.533E+00	-1.574E+02
	Li3	9.663E-01	2.953E-05	7.271E+00	7.271E+00	1.211E-02	7.271E+00	2.034E+00	1.985E+00	4.898E-02	-2.348E+01
**2.4**	N1	9.416E-03	-2.072E-05	5.457E+01	5.457E+01	6.101E-01	5.457E+01	6.991E+00	5.462E+00	1.528E+00	-1.565E+02
	N2	9.425E-03	-1.131E-05	5.457E+01	5.457E+01	6.101E-01	5.457E+01	6.991E+00	5.462E+00	1.528E+00	-1.565E+02
	Li3	9.811E-01	1.196E-05	7.243E+00	7.243E+00	1.198E-02	7.243E+00	2.019E+00	1.994E+00	2.477E-02	-2.224E+01
**3.0**	N1	3.367E-03	-4.633E-06	5.456E+01	5.456E+01	6.167E-01	5.456E+01	6.997E+00	5.471E+00	1.525E+00	-1.556E+02
	N2	3.383E-03	1.057E-05	5.456E+01	5.456E+01	6.166E-01	5.456E+01	6.997E+00	5.471E+00	1.525E+00	-1.556E+02
	Li3	9.933E-01	3.396E-06	7.231E+00	7.231E+00	6.473E-03	7.231E+00	2.007E+00	1.999E+00	8.112E-03	-2.100E+01
**5.0**	N1	4.275E-05	2.948E-06	5.456E+01	5.456E+01	6.221E-01	5.456E+01	7.000E+00	5.477E+00	1.523E+00	-1.542E+02
	N2	4.017E-05	-3.114E-06	5.456E+01	5.456E+01	6.220E-01	5.456E+01	7.000E+00	5.477E+00	1.523E+00	-1.542E+02
	Li3	9.999E-01	4.961E-06	7.228E+00	7.228E+00	8.772E-05	7.228E+00	2.000E+00	2.000E+00	8.928E-05	-1.905E+01

Table S5. Critical points (CP) properties for the N_2-Cs^+ system at 2.0. 2.4, 3.0 and 5.0 Å

**N_2-Cs^+ distance (Å)**	**CP**	**Type**	ρ $(electrons/{a_0}^3)$	$\rho_{nucleus}$ $(electrons/{a_0}^3)$	∇^2_{BCP} $(electrons/{a_0}^5)$	ϵ	**Bond radius GBL (a_0)**
	CP1	(3,-3) NACP N1	2.027E+02	2.027E+02	-	-	-
	CP2	(3,-3) NACP N2	2.027E+02	2.027E+02	-	-	-
**2.5**	CP3	(3,-3) NACP Cs3	3.507E+05	3.507E+05	-	-	-
	CP4	(3,-1) BCP N2 N1	7.192E-01	-	-2.844E+00	1.955E-02	2.062E+00
	CP5	(3,-1) BCP Cs3 N2	4.429E-02	-	1.783E-01	1.975E+00	4.725E+00
	CP1	(3,-3) NACP N1	2.027E+02	2.027E+02	-	-	-
	CP2	(3,-3) NACP N2	2.027E+02	2.027E+02	-	-	-
**2.8**	CP3	(3,-3) NACP Cs3	3.507E+05	3.507E+05	-	-	-
	CP4	(3,-1) BCP N2 N1	7.201E-01	-	-2.874E+00	5.119E-03	2.062E+00
	CP5	(3,-1) BCP Cs3 N2	2.473E-02	-	9.719E-02	1.349E+00	5.292E+00
	CP1	(3,-3) NACP N1	2.027E+02	2.027E+02	-	-	-
	CP2	(3,-3) NACP N2	2.027E+02	2.027E+02	-	-	-
**3.0**	CP3	(3,-3) NACP Cs3	3.507E+05	3.507E+05	-	-	-
	CP4	(3,-1) BCP N2 N1	7.204E-01	-	-2.884E+00	5.416E-05	2.062E+00
	CP5	(3,-1) BCP Cs3 N2	1.661E-02	-	6.575E-02	1.175E+00	5.670E+00
	CP1	(3,-3) NACP N1	2.027E+02	2.027E+02	-	-	-
	CP2	(3,-3) NACP N2	2.027E+02	2.027E+02	-	-	-
**5.0**	CP3	(3,-3) NACP Cs3	3.507E+05	3.507E+05	-	-	-
	CP4	(3,-1) BCP N2 N1	7.199E-01	-	-2.883E+00	2.193E-03	2.062E+00
	CP5	(3,-1) BCP Cs3 N2	2.585E-04	-	9.885E-04	4.256E-01	9.449E+00

Table S6. Properties from the integration of the atomic basins for the N_2-Cs^+ system at 2.5, 2.8, 3.0 and 5.0 Å

**N_2-Cs^+ distance (Å)**	**Atom A**	**q(A)**	L	K	K_{scaled}	μ **(intraatomic)**	**G**	**N(A)**	**LI**	**DI**	**V_{ne}(A,A)**
	N1	1.111E-02	-3.477E-05	5.477E+01	5.477E+01	5.945E-01	5.477E+01	6.989E+00	5.410E+00	1.579E+00	-2.317E+02
**2.5**	N2	1.113E-02	-1.107E-05	5.477E+01	5.477E+01	5.945E-01	5.477E+01	6.989E+00	5.410E+00	1.579E+00	-2.317E+02
	Cs3	9.777E-01	8.899E-06	8.794E+03	8.794E+03	2.629E-01	8.794E+03	5.402E+01	5.383E+01	1.963E-01	-1.925E+04
	N1	8.643E-03	-5.104E-05	5.472E+01	5.472E+01	6.049E-01	5.472E+01	6.991E+00	5.433E+00	1.497E+00	-2.235E+02
**2.8**	N2	8.719E-03	3.107E-05	5.472E+01	5.472E+01	6.050E-01	5.472E+01	6.991E+00	5.433E+00	1.558E+00	-2.235E+02
	Cs3	9.826E-01	3.852E-06	8.794E+03	8.794E+03	1.297E-01	8.794E+03	5.402E+01	5.390E+01	6.068E-02	-1.923E+04
	N1	7.181E-03	1.485E-05	5.471E+01	5.471E+01	6.100E-01	5.471E+01	6.993E+00	5.445E+00	1.548E+00	-2.189E+02
**3.0**	N2	7.145E-03	-2.395E-05	5.471E+01	5.471E+01	6.100E-01	5.471E+01	6.993E+00	5.445E+00	1.548E+00	-2.189E+02
	Cs3	9.857E-01	3.526E-06	8.794E+03	8.794E+03	7.743E-02	8.794E+03	5.401E+01	5.393E+01	8.792E-02	-1.922E+04
	N1	3.026E-04	2.949E-07	5.469E+01	5.469E+01	6.247E-01	5.469E+01	7.000E+00	5.476E+00	1.524E+00	-1.926E+02
**5.0**	N2	3.012E-04	-2.890E-06	5.469E+01	5.469E+01	6.247E-01	5.469E+01	7.000E+00	5.476E+00	1.524E+00	-1.926E+02
	Cs3	9.9940005400E-01	1.310E-06	8.794E+03	8.794E+03	3.230E-04	8.794E+03	5.400E+01	5.400E+01	1.178E-03	-1.917E+04

ELF analysis for each cation species

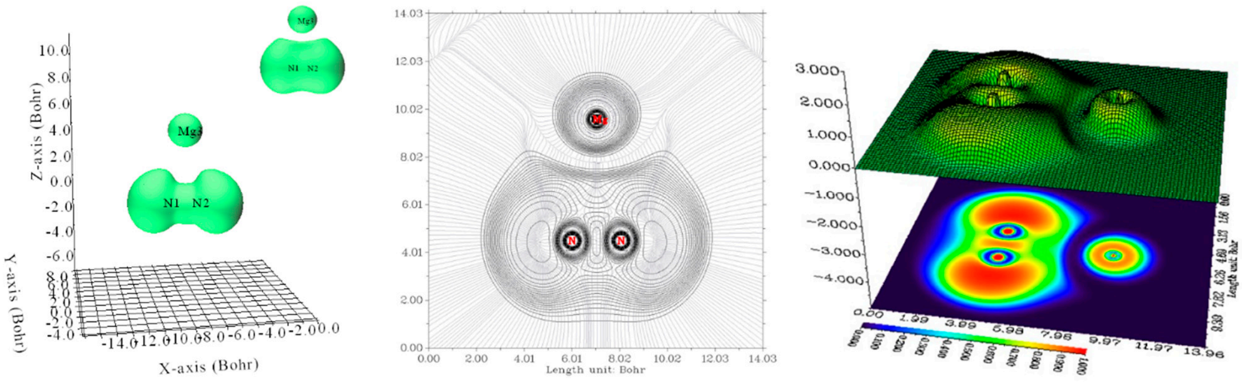

Figure S2. Effect of magnesium cation on the electron density of N_2 using ELF analysis

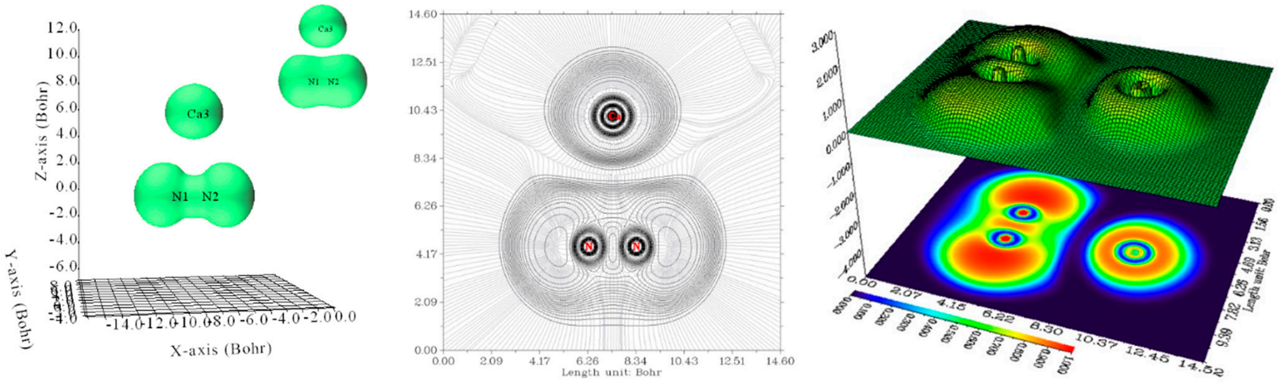

Figure S3. Effect of calcium cation on the electron density of N_2 using ELF analysis

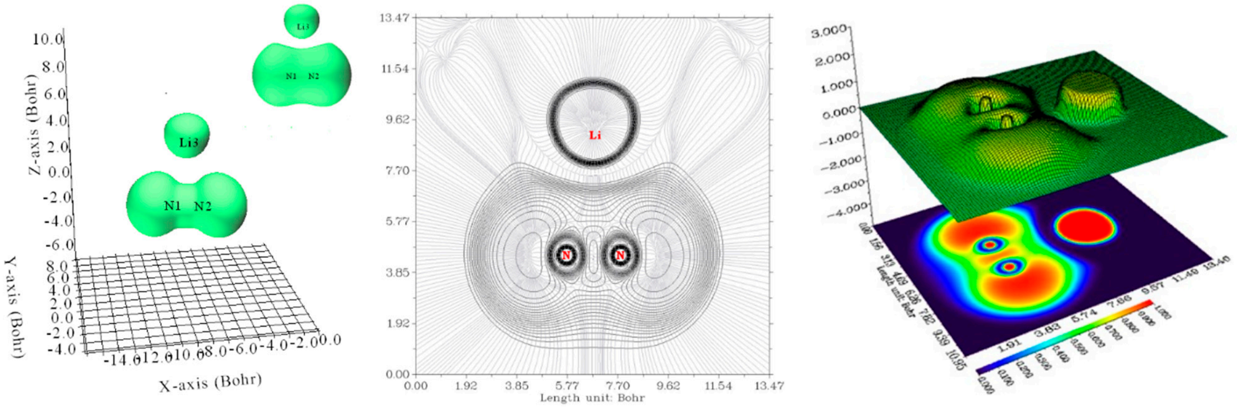

Figure S4. Effect of lithium cation on the electron density of N_2 using ELF analysis

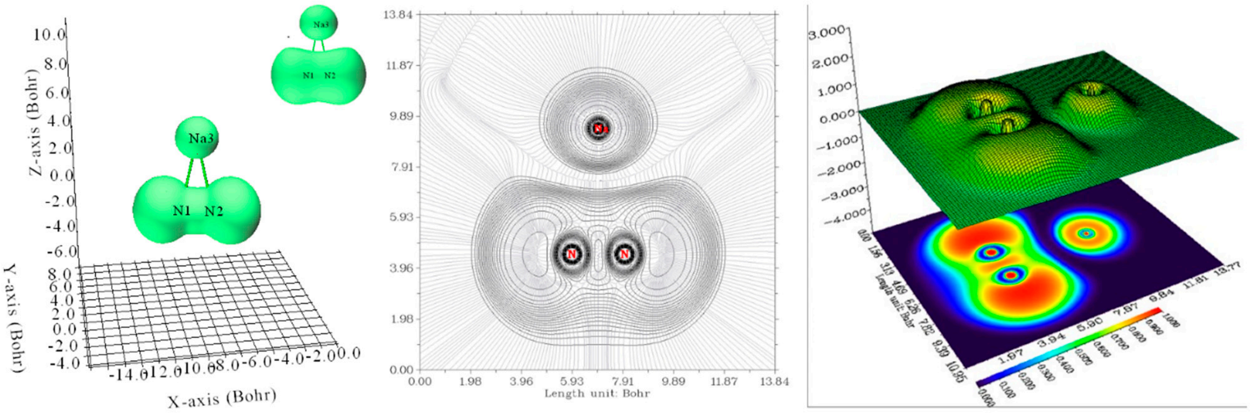

Figure S5. Effect of sodium cation on the electron density of N_2 using ELF analysis

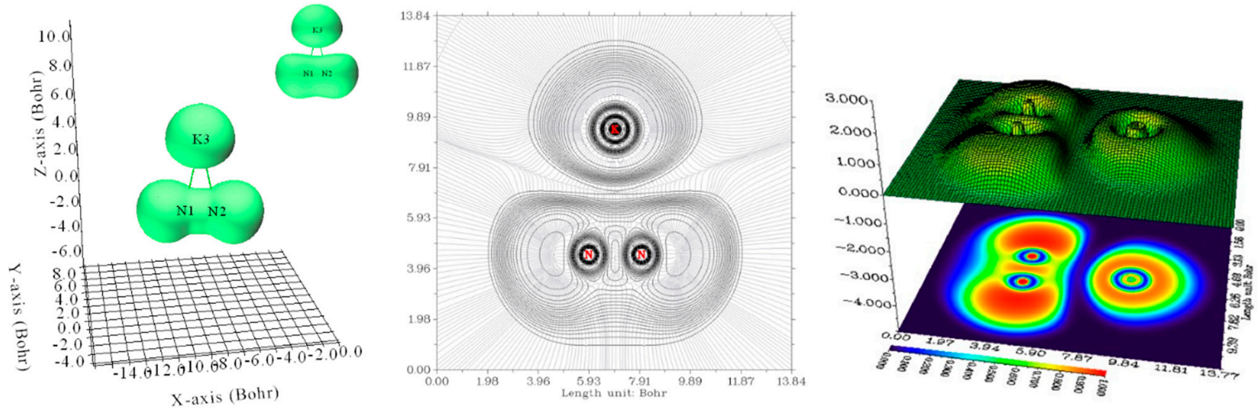

Figure S6. Effect of potassium cation on the electron density of N_2 using ELF analysis

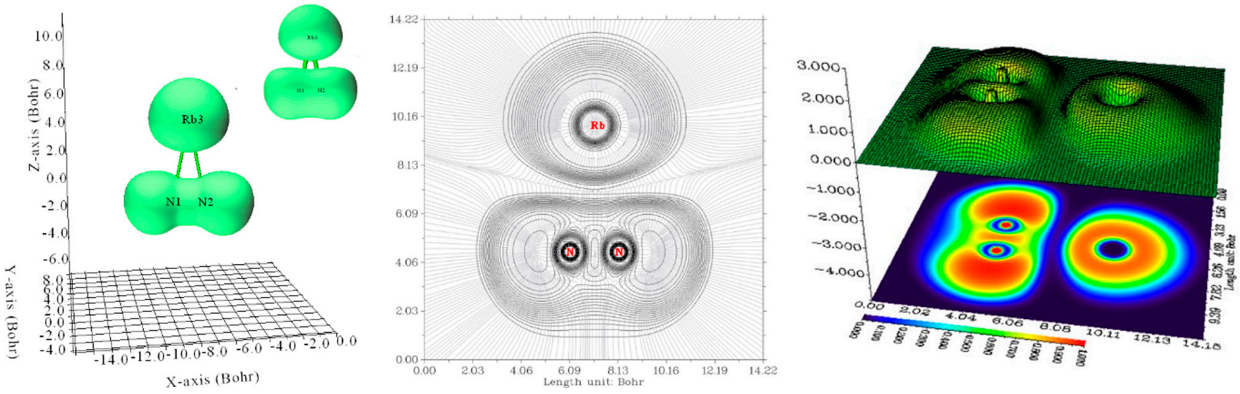

Figure S7. Effect of rubidium cation on the electron density of N_2 using ELF analysis

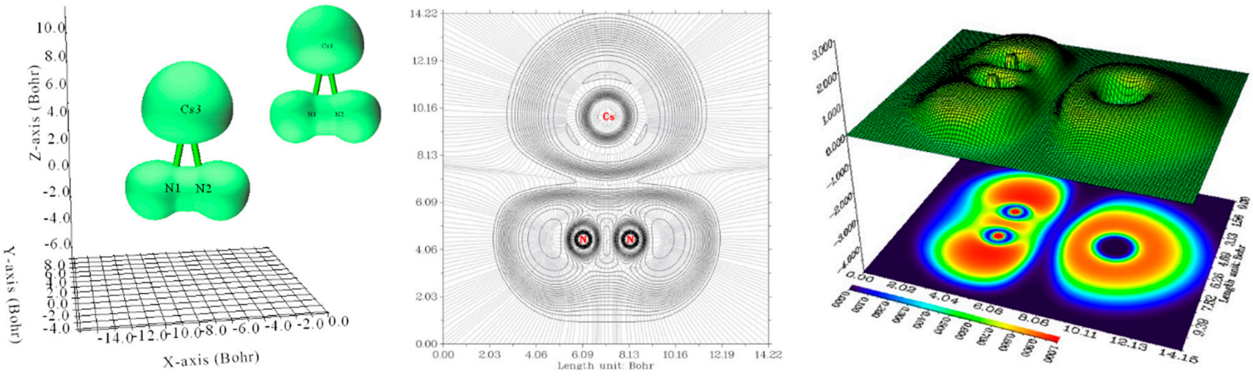

Figure S8. Effect of cesium cation on the electron density of N_2 using ELF analysis

MOs of the systems N_2-M^{n+}

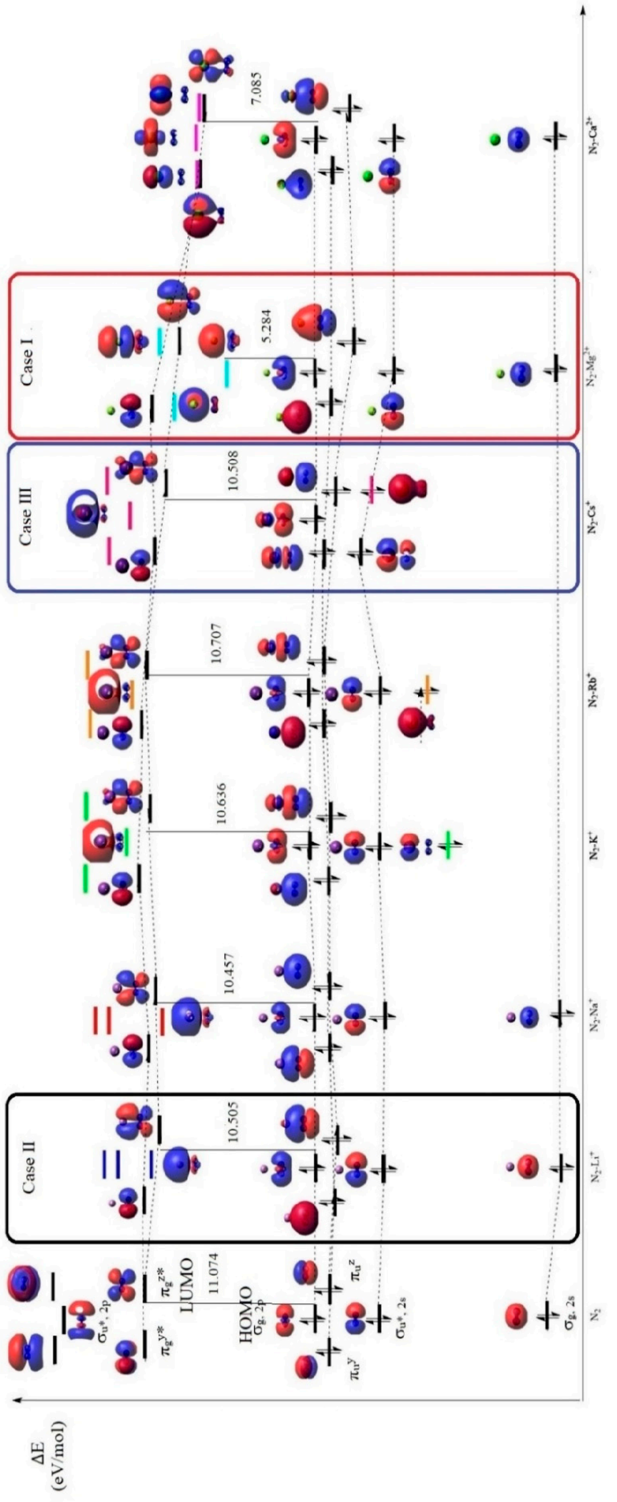

- *Figure S9. Overall MOs diagram for each cationic species. Case I represented by magnesium cation, Case II represented by lithium cation and Case III represented by cesium cation.*

Table S7. Non-occupied Cation AOs contribution to Bonding MOs of the system N_2-M^{n+}.

Cation	Electron conf.	HOMO	HOMO-1	HOMO-2	HOMO-3	HOMO-4
**Na^+**	$1s^22s^22p^6$	7s: 0.03; 8s: 0.02 $4p_z$:0.022; $5p_z$: 0.027 $6d_{z^2}$: 0.017	$4p_y$: 0.01	5s: 0.03; 7s: 0.075	-	-
**K^+**	$1s^22s^22p^6$ $3s^23p^6$	7s: 0.015; 8s: 0.015; 9s:0.014; 10s: 0.03; $5p_z$: 0.25; $7d_{z^2}$: 0.014	$6d_{yz}$: 0.012	5s: 0.02; 7s: 0.06; 8s: 0.026; 9s: 0.047; 10s: 0.017; $5d_{z^2}$:0.016; $6d_{z^2}$:0.022	-	$4p_x$:0.092; $4p_y$: 0.012; $5p_x$: 0.016 $6p_x$: 0.01
**Rb^+**	[Kr]$3d^{10}4s^24p^6$	$5p_z$: 0.022	4dyz: 0.011	6s: 0.019 $4d_{z^2}$: 0.014	-	-
**Cs^+**	[Rn]$4d^{10}5s^25p^6$	$6d_{z^2}$: 0.01	-	-	-	-
**Mg^+**	$1s^22s^22p^6$	5s: 0.013; 7s :0.023 8s:0.031; 9s: 0.012 $5p_z$: 0.026 $7d_{z^2}$: 0.01	$4p_y$: 0.013 $5p_y$: 0.015 $6d_{yz}$: 0.016 $4f_1$: 0.01	4s: 0.016; 5s: 0.044; 6s: 0.039 7s:0.138; 8s: 0.03; $4p_z$: 0.068; $5p_z$: 0.028; $6d_{z^2}$: 0.03	$4p_x$: 0.01 $6d_{xz}$: 0.012	7s:0.0092; $4p_z$: 0.0079
**Ca^+**	$1s^22s^22p^6$ $3s^23p^6$	7s: 0.012; 8s: 0.010 9s: 0.011; 10s: 0.025 $3p_z$:0.012; $5p_z$:0.017 $6p_z$:0.016; $7d_{z^2}$: 0.011	$5p_y$: 0.017 $5d_{yz}$:0.018	4s:0.012; 5s:0.017; 7s: 0.046 8s: 0.037; 9s: 0.1219; 10s: 0.042; $4p_z$: 0.019; $5p_z$: 0.051; $6p_z$: 0.019 $3d_{z^2}$: 0.022; $4d_{z^2}$: 0.028; $5d_{z^2}$: 0.039	$4d_{xz}$: 0.0076 $5d_{xz}$: 0.011	9s: 0.01

Optimal performance of cations for activating N_2

Table S8. N_2–M^{n+} system interactions at which the cations exercise their maximum activity for each case in Side-On type coordination.

Cation	N_2 – M^{n+} distance (Å)	MBO
**Mg^{2+}**	2.7	2.5707
**Ca^{2+}**	3.0	2.602
**Rb^+**	2.8	2.6247
**Cs^+**	2.8	2.6318
**Li^+**	2.4	2.6833
**Na^+**	2.6	2.6839
**K^+**	2.6	2.6859
